# Identification of a gene regulatory network associated with prion replication

**DOI:** 10.15252/embj.201387150

**Published:** 2014-05-19

**Authors:** Masue M Marbiah, Anna Harvey, Billy T West, Anais Louzolo, Priya Banerjee, Jack Alden, Anita Grigoriadis, Holger Hummerich, Ho-Man Kan, Ying Cai, George S Bloom, Parmjit Jat, John Collinge, Peter-Christian Klöhn

**Affiliations:** 1 MRC Prion Unit and Department of Neurodegenerative Disease, UCL Institute of Neurology, Queen SquareLondon, UK; 2 Department of Clinical Neuroscience, Karolinska InstituteStockholm, Sweden; 3 Biomedical Communications, Terrence Donnelly Health Sciences Complex, University of TorontoToronto, ON, Canada; 4 Breakthrough Breast Cancer Research Unit, Research Oncology, Guy's HospitalLondon, UK; 5 Department of Biology, University of VirginiaCharlottesville, VA, USA

**Keywords:** extracellular matrix, integrin, neurodegeneration, prion diseases, scrapie

## Abstract

Prions consist of aggregates of abnormal conformers of the cellular prion protein (PrP^C^). They propagate by recruiting host-encoded PrP^C^ although the critical interacting proteins and the reasons for the differences in susceptibility of distinct cell lines and populations are unknown. We derived a lineage of cell lines with markedly differing susceptibilities, unexplained by PrP^C^ expression differences, to identify such factors. Transcriptome analysis of prion-resistant revertants, isolated from highly susceptible cells, revealed a gene expression signature associated with susceptibility and modulated by differentiation. Several of these genes encode proteins with a role in extracellular matrix (ECM) remodelling, a compartment in which disease-related PrP is deposited. Silencing nine of these genes significantly increased susceptibility. Silencing of *Papss2* led to undersulphated heparan sulphate and increased PrP^C^ deposition at the ECM, concomitantly with increased prion propagation. Moreover, inhibition of fibronectin 1 binding to integrin α8 by RGD peptide inhibited metalloproteinases (MMP)-2/9 whilst increasing prion propagation. In summary, we have identified a gene regulatory network associated with prion propagation at the ECM and governed by the cellular differentiation state.

See also: **T Imberdis & DA Harris** (July 2014)

## Introduction

Transmissible spongiform encephalopathies or prion diseases are a family of fatal neurodegenerative diseases and include scrapie in sheep, bovine spongiform encephalopathy (BSE) in cattle, and Creutzfeldt-Jakob disease in humans. Prions, the transmissible agents, consist of aggregates of abnormal conformers of the cellular prion protein (PrP^C^), generally referred to as PrP^Sc^, and replicate in a self-perpetuating manner by conversion of host-encoded PrP^C^. Whilst the physiological role of PrP, a cell surface protein highly expressed in the central nervous system, is unclear, recent reports suggest that it may act as a receptor for amyloid beta (Aβ) in Alzheimer's disease (Lauren *et al*, 2009; Freir *et al*, 2011). To better understand molecular events that lead to prion neurodegeneration, it is critical to identify genetic factors that facilitate or impede prion replication. Coding polymorphisms within *Prnp*, the gene encoding PrP, are known to affect disease incubation times and susceptibility in human, mouse, and sheep (Hunter, 1997; Collinge, 2001). The most prominent example, codon 129 polymorphism in humans, has major disease-modifying effects (Collinge, 2001) and homozygosity for methionine at codon 129 confers susceptibility to variant CJD (vCJD) (Collinge *et al*, 1996; Collinge, 2005). However, significant differences in incubation times for scrapie in mice with the same *Prnp* genotype indicate a major role of PrP-independent genetic factors, and several genetic loci have been identified on different chromosomes (Carlson *et al*, 1993; Lloyd *et al*, 2001, 2009b). A number of knockout mice with disruptions in specific genes that were believed to affect prion replication did not show any discernible effect on the pathogenesis of prion disease (Tamguney *et al*, 2008). However, ablation of two genes, amyloid beta precursor protein (App) and interleukin-1 receptor type I (Il1r1), and transgenic overexpression of human superoxide dismutase 1 (SOD1) prolonged incubation times by 13, 16, and 19%, respectively (Tamguney *et al*, 2008). Our recent genome-wide association study identified two new common variants, the retinoic acid receptor beta (RARB) and stathmin-like 2 (STMN2) that are associated with risk of vCJD (Mead *et al*, 2009). An E3 ubiquitin ligase, HECTD2, was found to be associated with susceptibility to mouse and human prion disease (Lloyd *et al*, 2009a).

Mammalian cell lines have proven invaluable to investigate aspects of prion pathogenesis *in vitro,* such as infection and propagation (Race *et al*, 1987; Krammer *et al*, 2009; Marijanovic *et al*, 2009; Goold *et al*, 2011), prion strain selection (Li *et al*, 2010; Weissmann *et al*, 2011), and prion dissemination (Fevrier *et al*, 2004; Gousset *et al*, 2009). However, most PrP-expressing cell lines are resistant to prion infection, indicating that factors in addition to PrP are required to initiate and/or maintain chronic propagation of prions. To better understand the molecular underpinnings of neurodegeneration in prion diseases, we sought to study cognate susceptible and resistant cells, an approach that provides a unique opportunity to identify genetic factors that modulate prion replication.

We isolated rare prion-resistant revertants from highly susceptible mouse neuroblastoma N_2_a cells, determined the expression differences between resistant and susceptible cells, and identified a gene signature that was associated with inhibition of prion replication. Validation by RNA interference confirmed the inhibitory activities on prion replication of nine genes, most of which encode proteins expressed at plasma membrane level or at the ECM, a compartment where disease-associated PrP accumulates. Here, we use the term ‘disease-associated PrP’ (PrP^d^), rather than PrP^Sc^ as the latter is defined biochemically as proteinase K (PK)-resistant PrP, and it is now established that there are important PK-sensitive forms of disease-related PrP as well (Safar *et al*, 1998). We suggest that fibronectin, which is highly expressed in prion-resistant revertants, activates ECM-resident metalloproteinases in an integrin α8-dependent manner. Notably, inhibition of integrin α8 signalling by the fibronectin fragment inhibitor RGD increased prion susceptibility and inhibited metalloproteinase activation. We furthermore show that silencing of *Papss2*, a gene expressed in revertants, led to undersulfation of heparan sulphate, increased PrP^C^ deposition at the ECM and an increase in prion replication rates. Although the ECM has previously been implicated in modulating prion propagation (Caughey & Raymond, 1993b; Gabizon *et al*, 1993; Caughey *et al*, 1994), here we identify key genes involved in this process. The differential susceptibility of cell lines and different neuronal populations to prion infection has hitherto been unexplained, and these findings may be critical to understanding prion pathogenesis and selective vulnerability of different cell types to prion infection.

## Results

### Isolation of cognate prion-resistant revertants from highly susceptible cells

**Figure 1:**
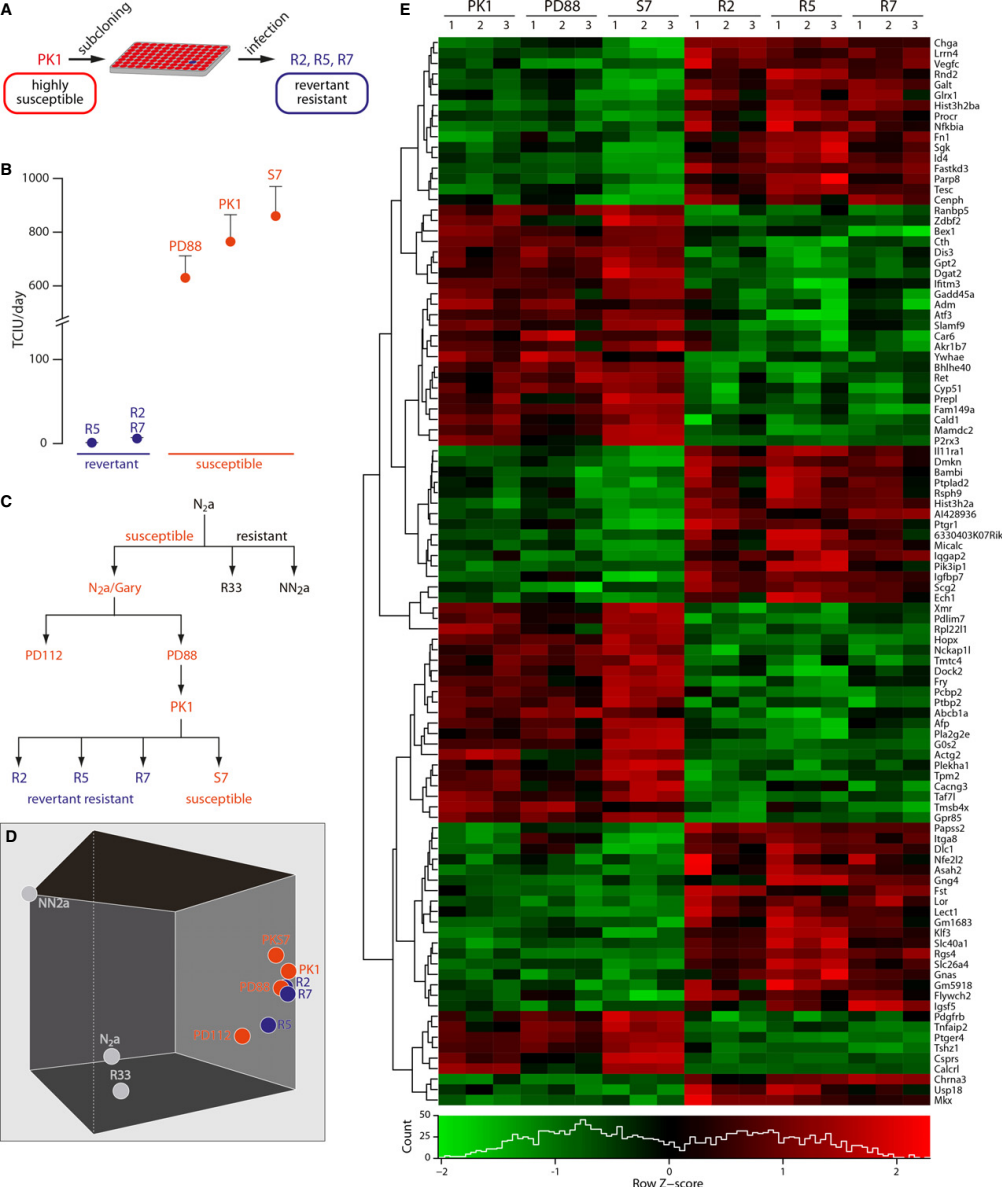
Characterisation of cognate prion-resistant revertants derived from highly susceptible cells Schematic for the isolation of prion-resistant revertants.Susceptible cells (S7, PK1, PD88) propagate prions 2–3 orders of magnitude faster than revertants. Prion propagation rates of cells infected with mouse RML prions are expressed as tissue culture infectious units (TCIU)/day.Lineage of susceptible and resistant cell clones isolated from parental N_2_a cells (grey: resistant, red: susceptible, blue: revertant resistant).Gene expression profiles of N_2_a cell clones were mapped onto a 3D transcript profile space after reducing dimensionality to three principal components. PK1-derived susceptible and revertant clones cluster around PK1 cells.Hierarchical clustering of genes differentially expressed between prion-resistant revertants and susceptible cells. Genes with a fold discovery rate (FDR) < 0.01 and a fold difference of at least two were included. Right legend: gene names, columns: samples of three biological repeats. Colour intensities based on expression level of genes as specified by the bar code on the bottom. Green: low-intensity values, red: high-intensity values, black: no change. Dendrogram cluster analysis on the left side.

Whilst most PrP-expressing neuronal cell lines are resistant to prions, subclones of otherwise poorly permissive cell lines showed marked differences in susceptibility to prion propagation (Bosque & Prusiner, 2000; Enari *et al*, 2001; Klohn *et al*, 2003; Mahal *et al*, 2007). After extensive subcloning, we derived PK1 cells, a mouse neuroblastoma cell line highly permissive to mouse RML prions (Fig1A and C), which we used to develop a sensitive cell-based prion bioassay, the Scrapie Cell Assay (SCA) (Klohn *et al*, 2003). We reasoned that the isolation of prion-resistant revertants from highly susceptible PK1 cells may allow the identification of genes associated with prion propagation by analysis of their respective transcriptomes. By determining prion propagation rates of a thousand PK1 subclones, three revertant clones (R2, R5, and R7) showed markedly reduced prion propagation rates when compared to susceptible PK1 cells (Fig1B). To further characterise the degree of kinship between cognate cell clones, we determined the global gene expression profiles of individual N_2_a clones depicted in Fig1C and subsequently reduced the complexity of data sets using principal component analysis (PCA) (Fig1D). When mapped onto a 3D transcript profile space, all PK1-derived subclones clustered around PK1 cells and were more distant from the parental N_2_a cells and their prion-resistant progeny, R33 and NN_2_a (Fig1C and D). Given the close kinship between PK1 and its progeny, we reasoned that gene expression analysis of cognate cell clones may be favourable to reduce the number of false-positive calls, that is, expression differences unrelated to the phenotype of prion susceptibility. We therefore excluded N_2_a, NN_2_a, and R33 cells and selected the six closely related cell clones (PD88, PK1, S7, R2, R5, and R7) for further analyses.

### Overexpression of PrP does not render revertants susceptible

Whilst accelerated disease progression was observed in prion-infected Tga20 mice which express PrP at about 10 times the wild-type level (Fischer *et al*, 1996), overexpression of PrP in a range of mouse N_2_a sublines did not increase susceptibility to mouse prions (Enari *et al*, 2001). To investigate whether the rate of prion propagation is a function of PrP expression, we stably overexpressed PrP in a variety of cell clones and determined their susceptibility to prions (Supplementary Table S1). To confirm that the expressed *Prnp* is functional, we used it to stably reconstitute *Prnp*-silenced PK1 cells (PK1 *Prnp-kd*) and challenged a heterogeneous pool of these cells with mouse RML prions. Whilst prion susceptibility was recovered by reconstituting PK1 *Prnp-kd* cells, revertants remained non-permissive to mouse RML prions after PrP overexpression. In addition, no significant increase in susceptibility of prion-permissive clones was observed at elevated PrP expression levels (Supplementary Table S1). To exclude the possibility that revertants express polymorphic *Prnp* and thus inhibit prion propagation by interference with the expressed *Prnp* transgene, we sequenced *Prnp* from representative PK1 clones. However, all PK1 subclones expressed *Prnp* allotype A (*Prnp*^*a*^), the allotype of the transgene. This indicates that PrP expression is necessary, but not sufficient to confer susceptibility to prion propagation.

### Differential gene expression between prion-resistant revertants and susceptible cells

We next determined differentially expressed genes between prion-resistant revertants (R2, R5, R7) and susceptible cells (PK1, S7, PD88) by non-parametric statistics using ‘Significance Analysis of Microarrays’ (SAM) (Tusher *et al*, 2001) and corrected raw values for multiple testing at high stringency with a false discovery rate (FDR) (Benjamini & Hochberg, 1995) < 0.01. Genes significantly expressed in prion-susceptible cells, and revertants are listed in Supplementary Table S2. Unsupervised hierarchical clustering clearly segregated genes from revertant and susceptible cells and revealed gene clusters with similar expression patterns as depicted in a heatmap (Fig1E). Functional annotation clustering was used to infer whether gene sets, annotated by gene ontology (GO) terms, were overrepresented in the set of differentially expressed genes, when compared to their representation in the whole mouse genome. Two highly enriched gene sets, cellular differentiation (18 genes) and development (16 genes), were identified in a list of 100 differentially expressed genes (Supplementary Table S3). Notably, a set of five genes with a role in negative regulation of differentiation were expressed in revertants (Supplementary Table S3). Consistent with this notion, revertant cells showed a less differentiated morphology than prion-susceptible cells (Supplementary Fig S1).

### A phenotypic switch from prion-resistant to susceptible cells reveals putative prion susceptibility genes

The enrichment of genes with a role in negative regulation of cell differentiation prompted us to test whether preincubation of revertants with retinoic acid (RA), a well-characterised differentiation agent, affected the rate of prion replication. Remarkably, preincubation of revertant clones with a single dose of 0.5 μM RA augmented the rate of prion replication by up to 40-fold as compared to vehicle alone (Supplementary Table S4). Under these conditions, the cellular morphology and the cell doubling rates of revertants were unaffected (Supplementary Fig S2). In contrast to this marked increase in susceptibility, the rate of prion propagation only doubled for the weakly susceptible clone PD88 and decreased for highly susceptible PK1 cells with a concomitant decrease in cell doubling (Supplementary Fig S2). These results suggest that cellular processes associated with the differentiation state of cells modulate susceptibility to prion propagation, in agreement with a nerve growth factor (NGF)-mediated increase in prion susceptibility of PC12 cells (Rubenstein *et al*, 1990).

**Figure 2:**
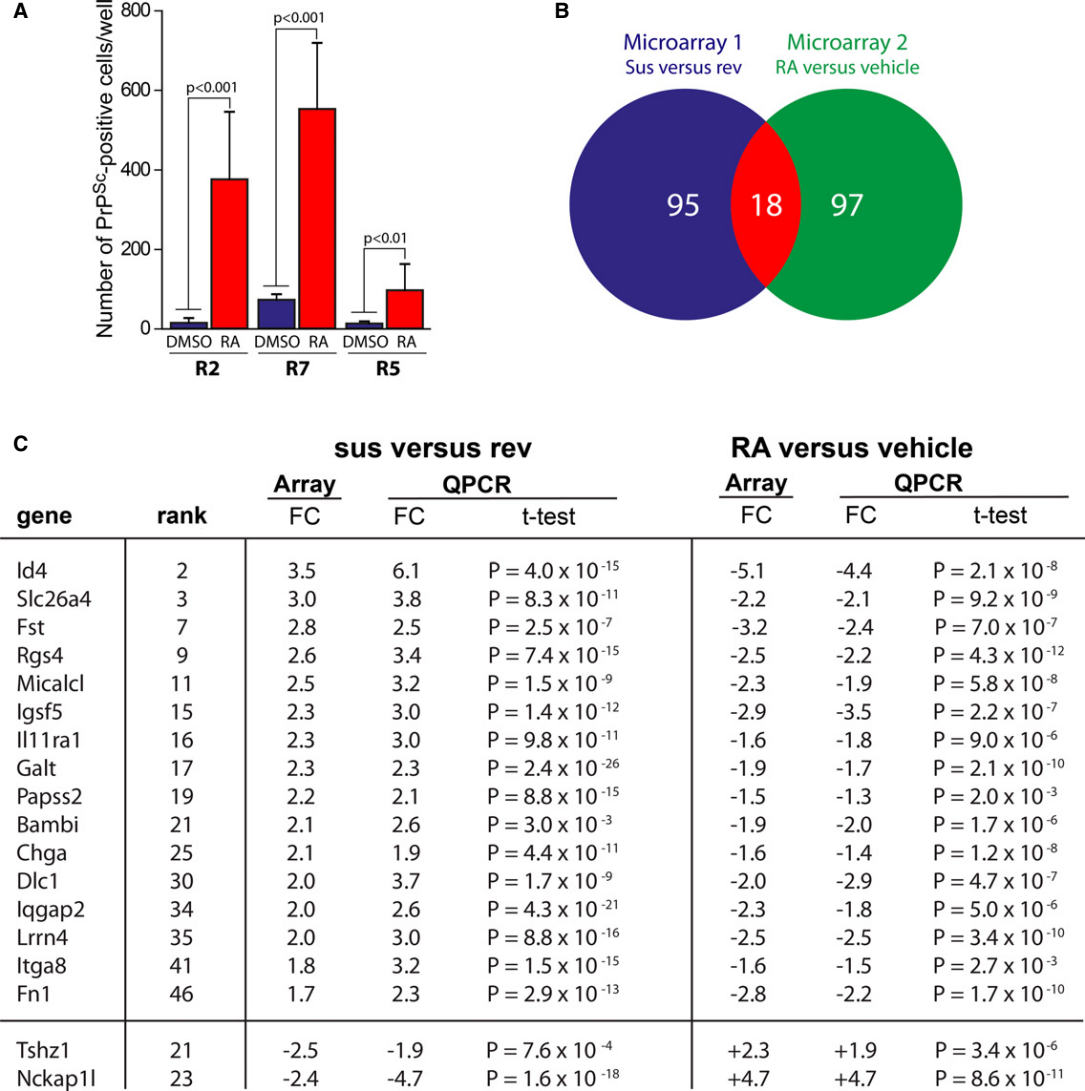
Identification of genes associated with a gain of prion susceptibility Increased susceptibilities of revertant cell clones R2, R5 and R7 after retinoic acid (RA) treatment, replotted from Supplementary Table S4 for clarity.A Venn diagram shows the relation between gene candidates derived from two independent microarray studies. The number of genes differentially expressed between susceptible and revertant clones (sus versus rev, Microarray 1) and revertant R7 cells in absence and presence of RA (RA versus vehicle, Microarray 2) is shown. The intersection in red represents 18 genes that are common to both gene candidate lists.Gene expression values of 18 putative prion susceptibility genes are shown. Fold expression changes (FC) of differentially expressed genes between susceptible and revertant cells and between mock- and RA-treated revertant R7 cells are shown for microarray (FDR < 0.01) and qPCR analysis, respectively, and ranked according to their FC values on microarray. The statistical significance of gene expression differences by qPCR are presented as discrete *P*-values (Student's *t*-test). Genes expressed in revertant and susceptible cells are represented as positive and negative FC values, respectively. Genes downregulated and upregulated upon RA treatment are presented as negative and positive FC values, respectively.

This RA-mediated phenotypic switch from prion-resistant to prion-susceptible cells (Supplementary Table S4) provided us with an experimental approach to identify gene candidates associated with a gain of prion susceptibility. We therefore determined genes that were differentially expressed in revertants in the presence and absence of RA (Supplementary Table S5) and compared this set of genes with the candidate list of previously identified differentially expressed genes between revertant and susceptible cells (Supplementary Table S2). Remarkably, eighteen of the previously identified genes were also differentially expressed upon RA treatment (Fig2B and C): sixteen genes expressed in revertants, but not in susceptible cells, were downregulated upon RA treatment, whereas two genes, Nckap1 l and Tshz1, downregulated in revertants, but expressed in susceptible cells, were induced in revertants under these conditions.

To validate the microarray data, we determined gene expression levels by quantitative real-time PCR (qPCR) using dual-labelled probes. Qualitative changes in gene expression values were fully confirmed with minor differences in gene expression levels (Fig2C). Together these data provide evidence for the identification of differentially expressed genes that are associated with prion susceptibility.

### Identification of a gene regulatory network associated with prion propagation

We next examined in a systematic gene silencing approach whether the loss of function of single candidates of the gene signature could recapitulate the gain of susceptibility observed upon RA differentiation of revertants (Fig2 and Supplementary Table S4).

**Figure 3:**
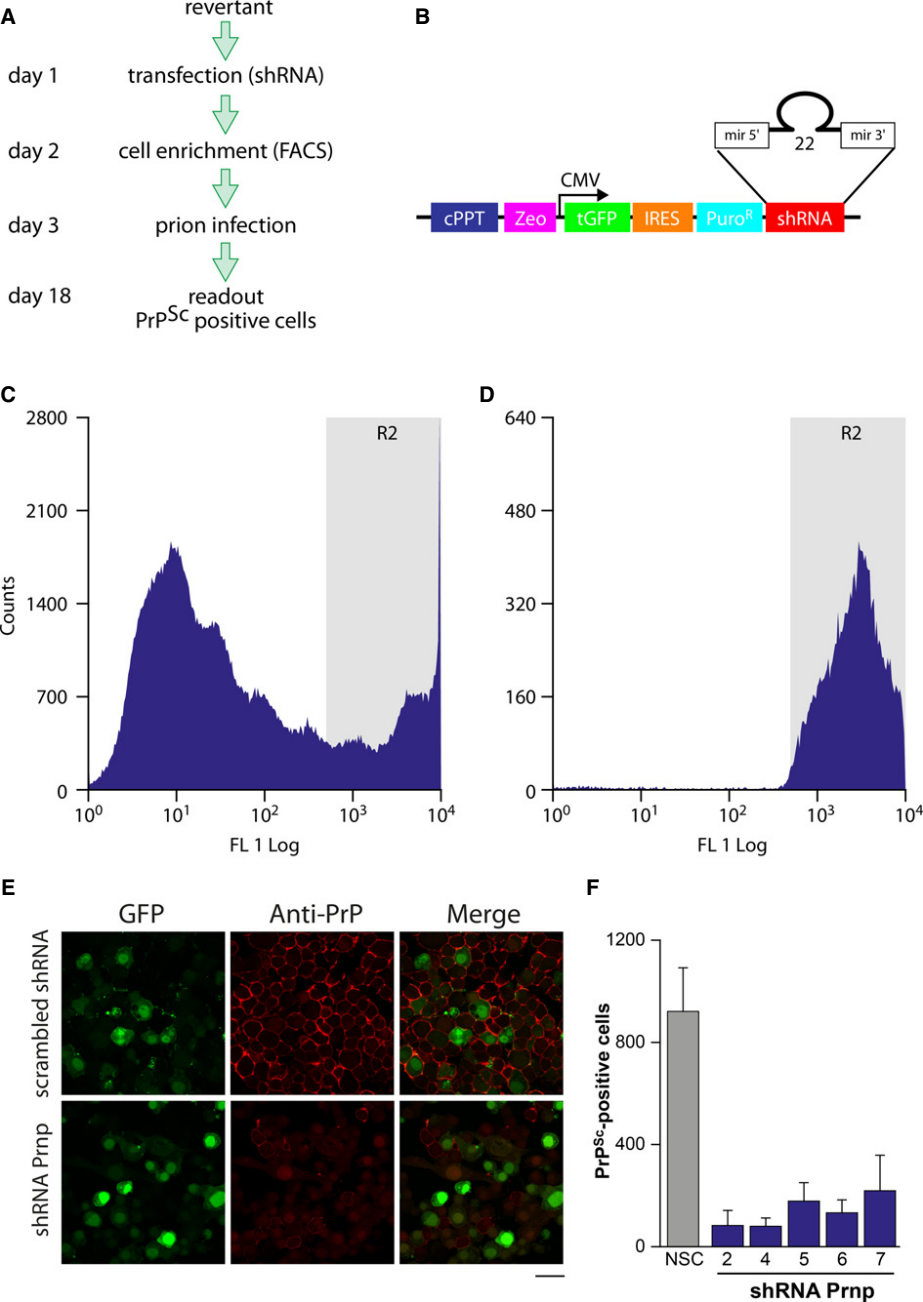
A gene silencing approach to validate genetic modifiers of prion propagation A Schematic representation of RNAi validation. B pGIPZ vector used for bicistronic expression of shRNA and GFP. C, D Enrichment of shRNA-expressing cells by gating highly GFP-positive cells using FACS. Fluorescence profiles of transfected cells before (C) or after (D) FACS enrichment of GPF-positive cells are shown. E Gene silencing of *Prnp* abrogates PrP protein expression at the plasma membrane. Revertant R7 cells were silenced with control shRNA (scrambled shRNA) and shRNA *Prnp*, enriched for GFP-positive cells and plated into chamber slides for immunofluorescence labelling. After 3 days cells were fixed and labelled with anti-PrP antibody ICSM18. Scale bar: 20 μm. F Transient gene silencing of *Prnp* inhibits prion propagation. Prion-susceptible PK1 cells were transfected with shRNA against *Prnp* or non-silencing control (NSC), enriched by flow cytometry, plated into 96-well plates at a cell density of 2 × 10^4^ cells/well and 24 h later infected with a 10^−5^ dilution of RML mouse prions. After three serial cell passages every 3–4 days, the number of PrP^Sc^-positive cells was determined by ELISA. Mean values ± SD are shown; a significant decrease in prion propagation was observed for all shRNAs tested (*P* < 0.01).

Due to the substantial number of gene candidates, we decided to transiently co-express short hairpin RNAs (shRNAs) alongside with green fluorescent protein (GFP) using an internal ribosomal entry (IRES)-based bicistronic vector (pGIPZ, Fig3B) and to enrich for GFP-expressing cells by fluorescent-activated cell sorting (FACS, Fig3A, C and D). To validate this assay, we transfected susceptible PK1 cells with five distinct shRNAs against *Prnp* and enriched from a heterogeneous pool of fluorescent cells (Fig3C) highly fluorescent cells in the 4th decade of the logarithmic fluorescence scale (Fig3D). As shown in cultured cells, the enrichment of GFP-fluorescent cells was associated with greatly reduced PrP expression levels (Fig3E). In a proof-of-concept experiment, we then demonstrated that transient *Prnp* silencing of prion-susceptible PK1 cells significantly reduced the rate of prion propagation (Fig3F). This enrichment procedure was used subsequently to examine whether gene silencing of each of our candidate genes affects prion replication rates.

Remarkably, a transition from a resistant to a susceptible phenotype could be recapitulated by single knockdown of any one of nine distinct genes: fibronectin 1 (*Fn1*), integrin α8 (*Itga8*), chromogranin A (*Chga*), IQ motif-containing GTPase-activating protein 2 (*Iqgap2*), interleukin 11 receptor, alpha chain 1 (*Il11ra1*), Micalc C-terminal like (*Micalcl*), regulator of G-protein signalling 4 (*Rgs4*), 3′-phosphoadenosine 5′-phosphosulphate synthase 2 (*Papss2*), and galactosyltransferase (*Galt*) (Table1). A complete list of gene silencing data is documented in Supplementary Table S6. In summary, these data verify the identification of a gene regulatory network associated with prion susceptibility.

**Table 1:** Gene silencing of distinct gene candidates in revertants is associated with increased prion susceptibility. Revertant R7 cells transiently expressing shRNA against distinct gene candidates using the bicistronic vector pGIPZ were enriched for highly GFP-fluorescent cells and subsequently infected with a 2 × 10^−5^ dilution of RML mouse prions. Rates of prion propagation were determined by SCA and normalised against cells transfected with non-silencing control vectors (NSC GIPZ). Relative rates of prion propagation expressed as fold change (FC) to controls (NSC) ± SD for at least three independent experiments are shown. The level of gene knockdown (% kd) was determined as described in Materials and Methods.

Gene symbol	*shRNA* construct	Rel. rate of prion propagation			Gene silencing	
		FC	SD	*t*-test	% kd	SD
*Chga*	*shRNA*-*Chga*.1	4.41	1.36	9.8 × 10^−7^	58	12
	*shRNA*-*Chga*.2	4.30	0.80	4.3 × 10^−8^	55	21
*Iqgap2*	*shRNA*-*Iqgap2*.4	5.65	1.07	1.3 × 10^−17^	55	27
*Fn1*	*shRNA*-*Fn1*.2	3.15	0.66	5.6 × 10^−5^	72	12
	*shRNA*-*Fn1*.6	3.39	0.42	3.5 × 10^−10^	89	17
*Itga8*	*shRNA-Itga8.2*	2.70	0.12	2.8 × 10^−9^	85	13
*IL11ra1*	*shRNA*-*IL11ra1*.1	2.46	0.92	5.6 × 10^−5^	54	8
	*shRNA*-*IL11ra1*.2	3.15	0.79	2.6 × 10^−7^	84	18
*Micalcl*	*shRNA*-*Micalcl*.1	2.85	0.59	5.9 × 10^−9^	95	15
	*shRNA*-*Micalcl*.3	2.47	0.16	9.2 × 10^−6^	83	16
*Rgs4*	*shRNA*-*Rgs4*.5	3.44	0.99	1.5 × 10^−7^	80	16
	*shRNA*-*Rgs4*.7	2.91	0.27	1.7 × 10^−8^	65	17
*Papss2*	*shRNA*-*Papss2*.1	2.32	0.13	1.6 × 10^−9^	48	9
	*shRNA*-*Papss2*.2	2.36	0.30	4.7 × 10^−9^	70	17
*Galt*	*shRNA*-*Galt*.1	1.60	0.32	2.2 × 10^−3^	42	20
	*shRNA*-*Galt*.4	1.82	0.50	2.5 × 10^−5^	41	17

Since the identified gene candidates were also expressed in susceptible cells, albeit at much lower expression levels, we examined whether gene knockdown in these cells might enhance their prion propagation kinetics further. Since S7, the cell clone with the fastest kinetics of prion propagation (Fig1B), showed poor transfection efficiencies with the pGIPZ vector, we used instead small inhibitory RNAs (siRNAs) to transiently silence gene expression with no subsequent cell enrichment to address this question. Remarkably, gene silencing of *Fn1*,*Micalcl* and *Papss2* significantly increased the rate of prion propagation by about twofold in S7 cells (Supplementary Table S7). Of note, knockdown of *Nckap1l*, a gene highly expressed in susceptible cells and overexpressed in revertants by fivefold after RA treatment (Fig2), significantly reduced prion susceptibility of S7 cells. This result confirms *Nckap1l*, a gene that was shown to be differentially expressed in brains of prion-diseased mice (Hwang *et al*, 2009) as a prion susceptibility gene.

The increased susceptibility of revertants may be due to several factors, such as the uptake of prions, their transport to replication sites, and the steady-state rates of synthesis and degradation (Weissmann, 2004). To examine whether the identified genes affect the steady-state levels of prion turnover, we silenced gene candidates in chronically prion-infected cells (iS7) and determined relative changes of prion levels 3 days after transfection with siRNA or scrambled control RNA (Supplementary Table S8). Remarkably, a significant increase of prion conversion rates was determined for all genes.

To investigate whether the identified genes affect prion propagation in a strain-specific manner, we challenged R7 cells with 22L and determined changes in susceptibility (Supplementary Table S9). Whilst a trend to increased prion propagation rates was observed for all genes studied, except for Galt, statistically significant results were obtained for more than half of the genes, including *Fn1*,*Itga8*,*Papss2*,*Chga*,*Il11ra1,* and *Lrrn4*. We conclude that some of the identified genes may control prion susceptibility in a strain-independent manner.

To examine whether the increase in prion susceptibility by loss of gene function is restricted to N_2_a-derived cells, we silenced a selection of candidate genes in CAD5 cells, a cell line derived from CNS catecholaminergic-differentiated (CAD) cells (Mahal *et al*, 2007) prior to RML infection. Knockdown of four out of eight candidate genes (*Fn1, Galt, Il11ra1*, and *Itga8*) resulted in a significant increase in susceptibility (Supplementary Table S10), indicating that control of prion propagation by the identified genes is not restricted to N_2_a cells.

### Prion modifiers are expressed at the extracellular matrix and plasma membrane level

**Figure 4:**
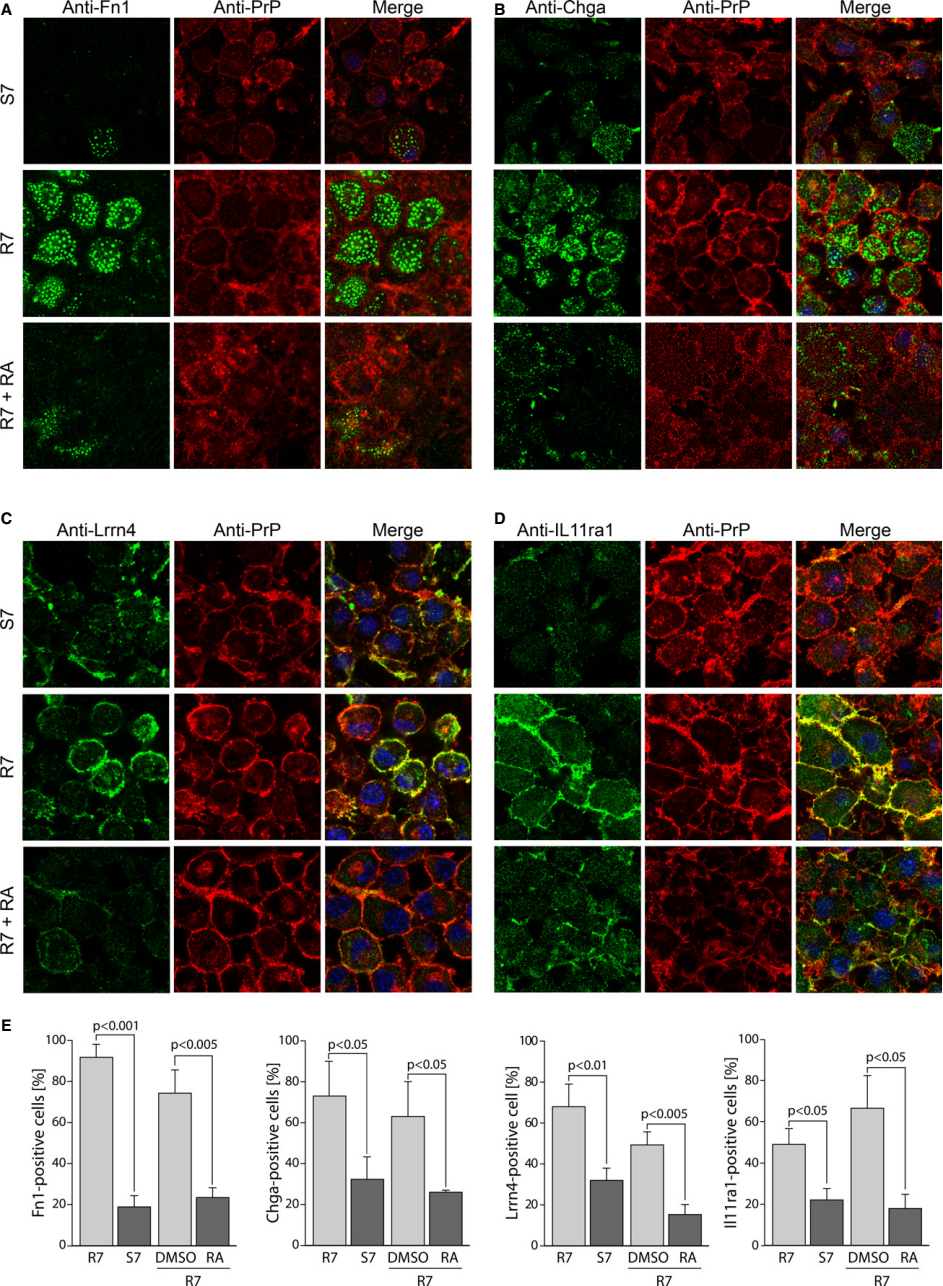
Prion modifiers are expressed at the extracellular matrix and plasma membrane level A–D Representative images of fixed and permeabilised cells labelled with antisera against (A) Fn1, (B) Chga, (C) Lrrn4, and (D) Il11ra1. Images for Lrrn4 (C) were collected at mid-cell level, all other images at ECM level. Differences in protein expression levels between S7, R7 cells and R7 cells in presence of RA, respectively, are shown and are in agreement with gene expression data (Fig2). E The relative abundance of cells positive for Fn1, Chga, Lrrn4, and Il11ra1 in susceptible S7 and revertant R7 cells, in presence and absence of RA, analysed using Volocity image analysis software as described in Methods are shown. Mean numbers ± SD for 20 frames are shown. The degree of colocalisation between candidate proteins and PrP was determined in R7 cells and is represented as Pearson's correlation coefficients (PCC): (A) 0.11 ± 0.04, (B) 0.20 ± 0.12, (C) 0.55 ± 0.09, and (D) 0.36 ± 0.14.

To characterise the subcellular location of prion modifier proteins, we sourced suitable commercial anti-rabbit antibodies and co-immunolabelled candidate proteins and PrP (ICSM18) in fixed and permeabilised S7 and R7 cells (Fig4). All co-labelling studies were conducted with highly cross-absorbed secondary antibodies to exclude cross-reactivity. Protein expression levels of Fn1, Chga, Lrrn4, and Il11ra1 were elevated in prion-resistant R7 compared to susceptible S7 cells as anticipated from the corresponding gene expression data (Fig2). Furthermore, the expression of candidate proteins in R7 was greatly reduced upon treatment with 0.5 μM RA (Fig4A–E). Fn1, a protein expressed at the extracellular matrix (ECM) with a major role in cell adhesion, migration, and differentiation, showed punctate, but no fibrillar structures, reminiscent of cells with defects in matrix assembly (Yoneda *et al*, 2007) (Fig4A). Similarly, Chga, a secretory protein with a role in regulation of secretory granule synthesis (Kim *et al*, 2001), was deposited at the ECM as depicted in R7 cells (Fig4B). Neither Fn1 nor Chga showed colocalisation with PrP at the ECM level as documented by their corresponding Pearson correlation coefficients (PCC, Fig4A and B). In contrast, Lrrn4 and Il11ra1, which were expressed at the ECM and the membrane level, showed partial colocalisation with PrP with PCC values of 0.55 ± 0.09 and 0.36 ± 0.14, respectively (Fig4C and D). An antibody against integrin α8 confirmed higher protein expression levels in R7 in comparison with S7 cells; however, no expression difference could be detected in presence and absence of RA (Supplementary Fig S3A), in agreement with gene expression levels (Fig2). Micalcl, a putative binding protein of extracellular signal-regulated kinase 2 (ERK2) (Miura & Imaki, 2008), was expressed at the ECM as shown by N-terminal fusion of Miclacl with YFP (Supplementary Fig S3B).

Iqgap2, a cytoskeletal scaffolding protein, was expressed at the membrane level (Supplementary Fig S3C). Since antibodies against PrP and Iqgap2 were both raised in mice, double-labelling experiments could not be performed. The protein subcellular location of Rgs4, Fst, Papss2, and Galt could not be determined due to the lack of specificity of commercial antibodies.

### Detection of aberrant PrP^d^ deposits at the ECM after delipidation with acetone

**Figure 5:**
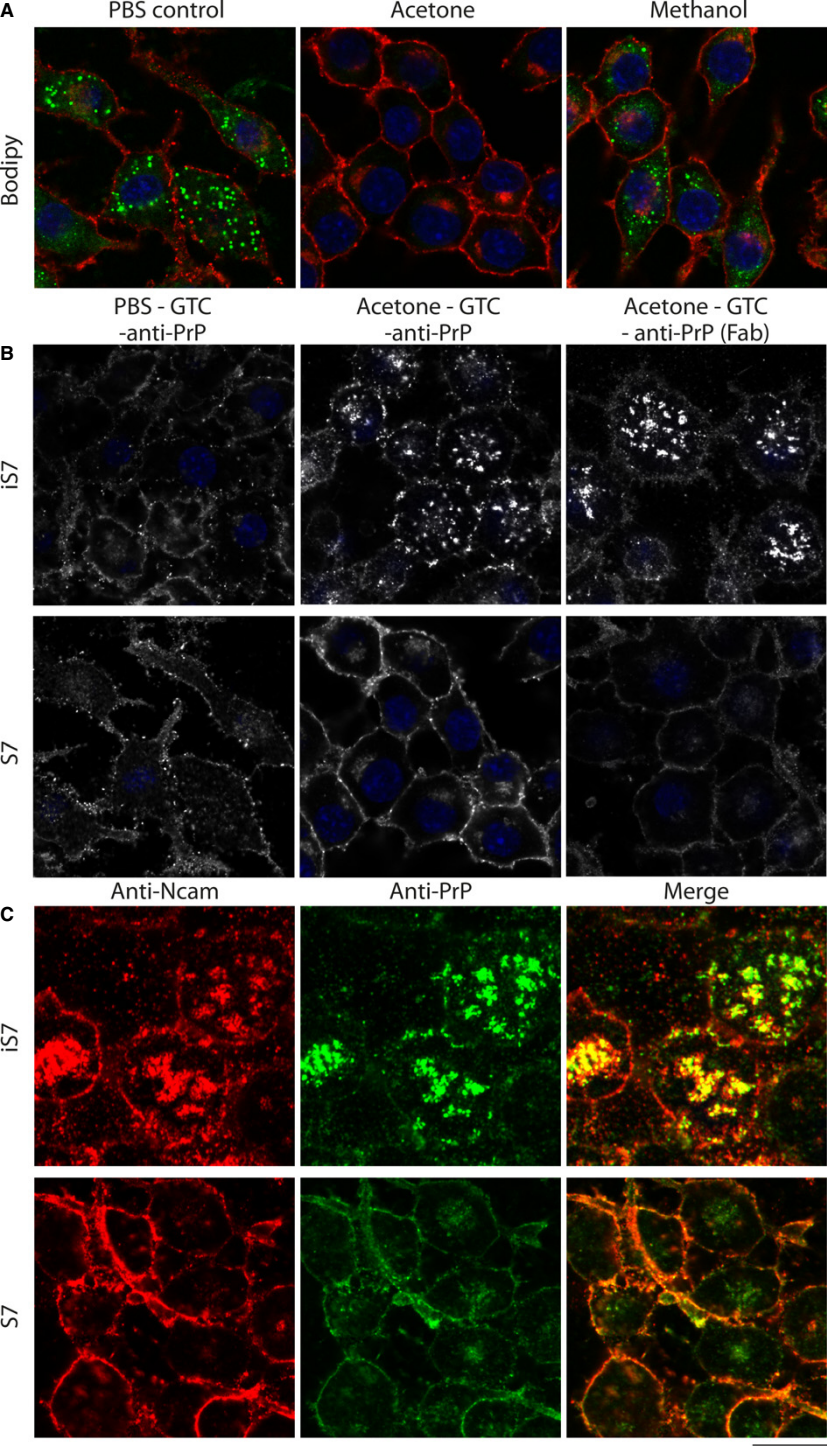
Aberrant deposition of PrP^d^ in the extracellular matrix of cells revealed after delipidation with acetone Susceptible S7 cells were labelled with 1 μM C1-BODIPY 500/510 (green label) for 12 h, fixed, and treated for 1 min with ice-cold acetone, methanol or PBS. Cells were counter-stained with ICSM18 (red label).Chronically infected iS7 cells and uninfected S7 cells were fixed, delipidated with acetone or treated with PBS. All samples were denatured with 3 M GTC and washed at least five times with PBS before labelling with anti-PrP antibody ICSM18 or ICSM18 Fab fragment (Fab).Infected iS7 and uninfected S7 cells were fixed, delipidated with acetone, and denatured with 3 M GTC prior to labelling with anti-NCAM and ICSM18 (anti-PrP). The degree of colocalisation, expressed as PCC values are 0.42 ± 0.08 for iS7 cells and 0.56 ± 0.08 for uninfected S7 cells. Data information: Scale bar, 20 μm.

To investigate how the expression of prion modifiers might interfere with prion formation on a subcellular level, we sought to determine PrP^d^ by immunofluorescence (IF) on formaldehyde-fixed cells according to established protocols (Veith *et al*, 2008; Marijanovic *et al*, 2009; Goold *et al*, 2011). However, whilst >95% of chronically infected S7 cells (iS7) were PrP^Sc^-positive on SCA, the proportion of cells with aberrant PrP^d^ deposits revealed by IF after guanidinium or formic acid treatment (Veith *et al*, 2008; Goold *et al*, 2011) did not exceed 20% in agreement with previous studies (Goold *et al*, 2011) (Supplementary Fig S4B). We reasoned that procedural differences between the two assay types might account for differences in the proportion of PrP^d^. To investigate whether heat-treatment of cells after transfer to Elispot plates during SCA (Klohn *et al*, 2003), a treatment known to cause membrane delipidation, may explain these inconsistencies, we treated fixed cells with delipidating solvents prior to immunolabelling with anti-PrP antibody ICSM18. Delipidation of fixed cells with acetone, a solvent that preferentially dissolves neutral lipids, such as triacylglycerols and cholesterol esters, but not with methanol a solvent that dissolves polar lipids, such as phospholipids and glycosphingolipids, quantitatively removed neutral lipids in cells, as evidenced by the loss of C1-BODIPY 500/510 fluorescence (Fig5A). C1-BODIPY-500/510 is a fatty acid analogue, which is deposited in triacylglyceride-rich lipid droplets. Similar results were obtained with BODIPY-cholesterol (Supplementary Fig S5). Strikingly, acetone pretreatment followed by denaturation with guanidinium thiocyanate (GTC) revealed PrP^d^ deposits at the basement membrane level of iS7 cells, a phenotype that was absent in uninfected cells (Fig5B). Similar labelling patterns were shown with Fab fragments of ICSM18, thus excluding the possibility of PrP redistribution upon binding of a divalent antibody (Fig5B). A colocalisation of PrP^d^ with neural cell adhesion molecule (NCAM) confirmed the deposition of PrP^d^ at the ECM (Fig5C, Supplementary Video S1). In contrast to the detection of abundant PrP^d^ deposits at the ECM following acetone and GTC treatment, PrP^d^ was detected predominantly in endosomal and perinuclear areas following formic acid or methanol/GTC treatment (Supplementary Fig S4A). Punctate PrP^d^ deposits in iS7 cells at the ECM are visible upon treatment with acetone and GTC, but are absent in controls (Supplementary Fig S4C). Abundant punctate PrP^d^-positive patches were also detected on membranes above ECM level in delipidated iS7 cells (Supplementary Fig S4D). The detection of PrP^d^ deposits following acetone delipidation and GTC treatment is not restricted to N2a cells as shown for chronically infected prion-permissive CAD5 cells (Mahal *et al*, 2007) (Supplementary Fig S4E). In summary, our results suggest that the cryptic ICSM18 epitope in PrP^d^ deposits at the ECM is masked by neutral lipids.

### Distinct phenotypes of prion-modulatory proteins at the ECM of chronically infected cells

**Figure 6:**
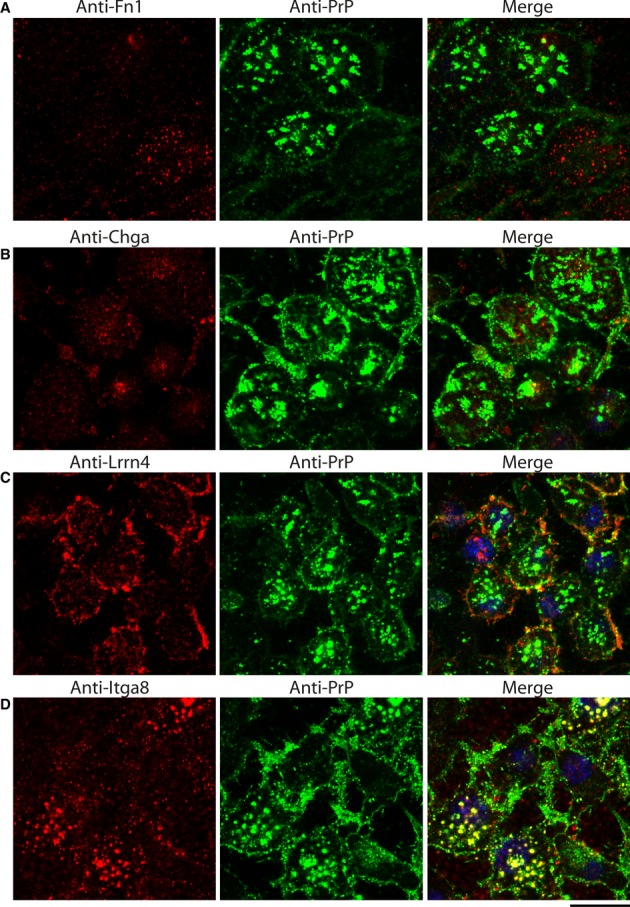
Distinct phenotypes of prion-modulatory proteins at the ECM of chronically infected cells A–D Chronically infected iS7 cells were fixed, delipidated with acetone, and denatured with 3 M GTC. Cells were then co-labelled with ICSM18 and (A) anti-Fn1, (B) anti-Chga, (C) anti-Lrrn4, and (D) anti-Itga8. After washing of primary antibodies with sterile PBS, cells were incubated with highly cross-absorbed anti-mouse (ICSM18) and anti-rabbit (all other antibodies) Alexa Fluor-conjugated secondary antibodies. Representative images are shown. PCC values: (A) 0.03 ± 0.03, (B) 0.08 ± 0.03, (C) 0.16 ± 0.09 and (D) 0.28 ± 0.07. Data information: Scale bar, 20 μm.

The detection of PrP^d^ deposits at the ECM of chronically infected cells now enabled us to investigate the subcellular distribution of prion-modulatory proteins in relation to aberrant PrP^d^. Remarkably, cells expressing Fn1 at the ECM level were completely devoid of PrP^d^ deposits (Fig6A), implying that Fn1 expression is negatively correlated with PrP^d^ deposition in susceptible iS7 cells. Similarly, Chga, which is poorly expressed in iS7 cells, does not colocalise with PrP (Fig6B). Lrrn4 was expressed in chronically infected cells, albeit with a low level of colocalisation with PrP at the plasma membrane (Fig6C). Of note, integrin α8 colocalised with aberrant PrP^d^ deposits at the ECM (Fig6D), but not at the plasma membrane (Supplementary Fig S3A).

### Disruption of integrin α8 signalling inhibits Fn1-mediated metalloproteinase activation and augments the rate of prion replication

**Figure 7:**
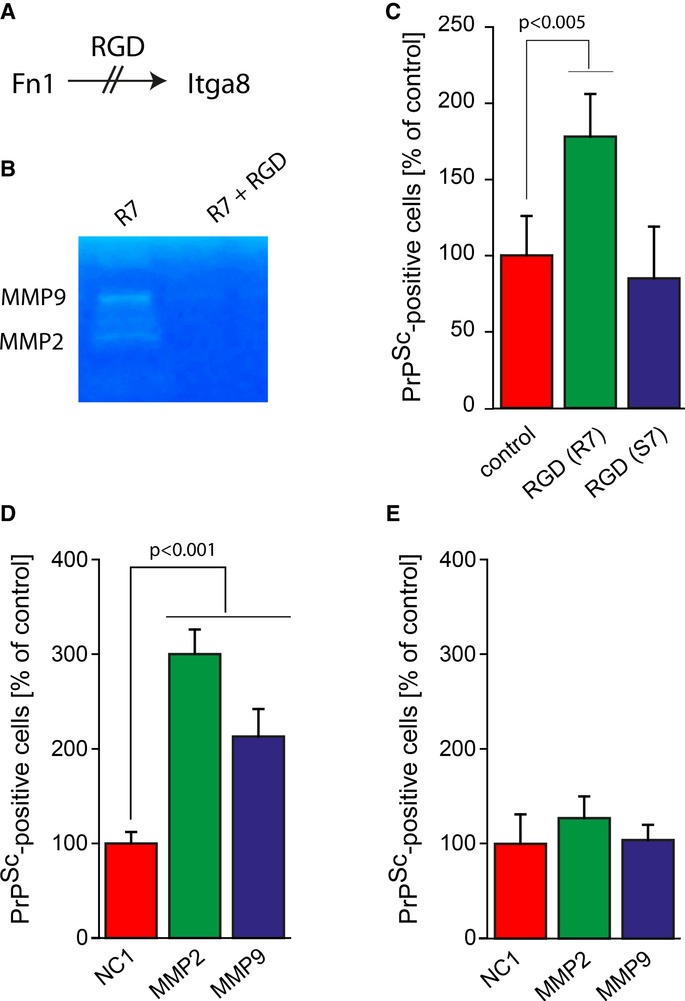
Disruption of integrin α8 signalling inhibits Fn1-mediated metalloproteinase activation and augments the rate of prion replication A Schematic representation of RGD effect. B Incubation of R7 cells with RGD peptide inhibits secretion of activated MMP2/9 as shown by gelatine zymography. C Preincubation with RGD of R7, but not S7 cells significantly increases the number of PrP^S^^c^-positive cells after prion infection. D, E Transient gene silencing of MMP2 and MMP9 using siRNA significantly increases the number of PrP^S^^c^-positive cells in R7 (D), but not in S7 (E) cells.

The question remained how the expression and deposition of secreted proteins might inhibit prion replication and aggregate formation at the ECM. Of note, RA-mediated remodelling of the ECM mimicked a gain of susceptibility, suggesting that matrix homeostasis and prion replication may be affected by common signalling pathways. Cellular differentiation is associated with ECM remodelling, regulation of matrix metalloproteinases (MMPs), reorganisation of the actin cytoskeleton, and changes in cell shape. These changes affect integrin signalling and the integrin-mediated crosstalk with growth factors. In our study, gene silencing of Itga8 and Fn1 was associated with a gain of susceptibility (Table1). To examine whether prion replication is affected by Fn1-mediated integrin signalling, we incubated revertants and susceptible cells with RGD, a peptide that blocks the interaction between Fn1 and integrins (Koivunen *et al*, 1995) (Fig7A). Whilst two integrins of the β1 subunit family, integrin α5 and integrin α8, harbour an RGD domain (Margadant *et al*, 2011), only the latter is expressed in susceptible and revertant cells. Remarkably, incubation of R7 cells with RGD inhibited secretion of activated MMP2 and MMP9 (Fig7B) and significantly increased the susceptibility of R7, but not of S7 cells (Fig7C). Furthermore, a loss of function of MMP2 and MMP9 significantly increased the number of PrP^Sc^-positive cells in R7 cells (Fig7D), but not in S7 cells (Fig7E). This argues that the integrin α8-dependent activation and secretion of MMP2/9 in revertant R7 cells are mediated by Fn1 and associated with an inhibitory environment for prion replication at the ECM.

### *Papss2* loss of function leads to undersulphation of heparan sulphate proteoglycans and augments prion susceptibility

**Figure 8:**
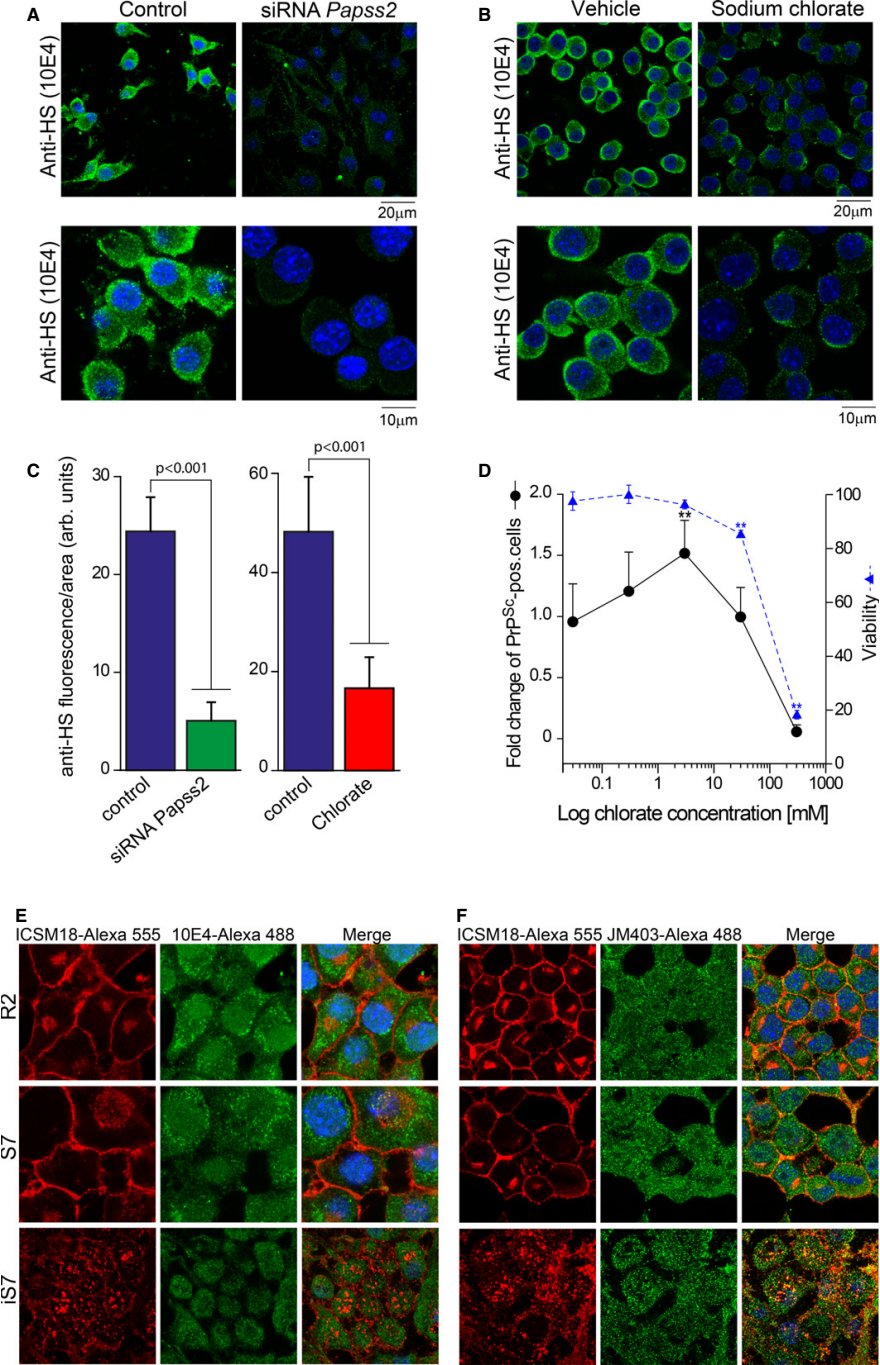
Loss of *Papss2* function leads to undersulphation of heparan sulphate proteoglycans A, B R2 cells were transfected with siRNA against *Papss2* and scrambled RNA control (A) or with 300 μM sodium chlorate and vehicle (PBS, (B)). After 3 days, cells were labelled with an anti-heparan sulphate (HS, 10E4) antibody and fluorescence intensities recorded at different magnifications (top and bottom panel). C Quantitative analysis of fluorescence intensities using Volocity. D Dose-response effects of sodium chlorate on the number of PrP^Sc^-positive cells in chronically infected cells (iS7) and cell viability, assessed by quantifying changes in cellular ATP levels using Ultra-Glo luciferase assay (Promega) in parallel experiments. Statistically significant differences (*P *< 0.001) between control and chlorate treatments are denoted (**). E, F No colocalisation between PrP and the 10E4 epitope (E) and between PrP and the JM403 epitope (F) was observed using covalently Alexa Fluor-conjugating antibodies ICSM18 and anti-HS in the cell types specified.

Papss2 (3′-phosphoadenosine-5′-phosphosulphate (PAPS) synthase 2), one of the principal enzymes required for the sulphation of extracellular matrix molecules (Wang *et al*, 2012), catalyses the synthesis of activated sulphate, PAPS, in cells. *Papss2* is expressed in revertants, and loss of function is associated with increased susceptibility (Table1, Supplementary Table S8). By using a sulphate-specific anti-heparan sulphate (HS) antibody (David *et al*, 1992), we show that loss of *Papss2* function in prion-resistant revertants leads to undersulphation of heparan sulphate proteoglycans (HSPGs, Fig8A). A similar effect was achieved by incubation of cells with sodium chlorate, an inhibitor of sulfurylase, required for the formation of PAPS (Fig8B). In agreement with loss of *Papss2* function in chronically prion-infected cells (Supplementary Table S8), the number of PrP^Sc^-positive cells significantly increased at 3 mM chlorate (Fig8D). The dose-response curve is biphasic due to a loss of cell viability at concentrations higher than 3 mM chlorate. Treatment of chronically infected cells with 30 mM chlorate in a previous study led to an inhibition of PrP^Sc^ accumulation (Ben Zaken *et al*, 2003). The discrepancy between this result and our study remain unexplained. To unambiguously examine whether the 10E4 epitope colocalises with PrP^C^, we covalently conjugated anti-HS (mouse IgM) and ICSM18 (mouse IgG1) with Alexa Fluor dyes (Fig8E). Our data suggest that the 10E4 epitope colocalises neither with PrP^C^ in S7 and R2 cells, nor with PrP^d^ deposits in chronically infected cells (Supplementary Table S11). In addition, a monoclonal antibody against N-unsubstituted heparan sulphate residues (JM403) (Vandenborn *et al*, 1992), which is unaffected by the sulphation state of HSPGs (Supplementary Fig S6), was used to test whether PrP^C^ colocalises with HSPGs. As shown in Fig8F and Supplementary Table S11, no colocalisation was observed between JM403 epitope and PrP in uninfected and chronically infected cells.

### Phenotypic differences in PrP^C^ densities at the ECM upon loss of *Papss2* and *Fn1* function

**Figure 9:**
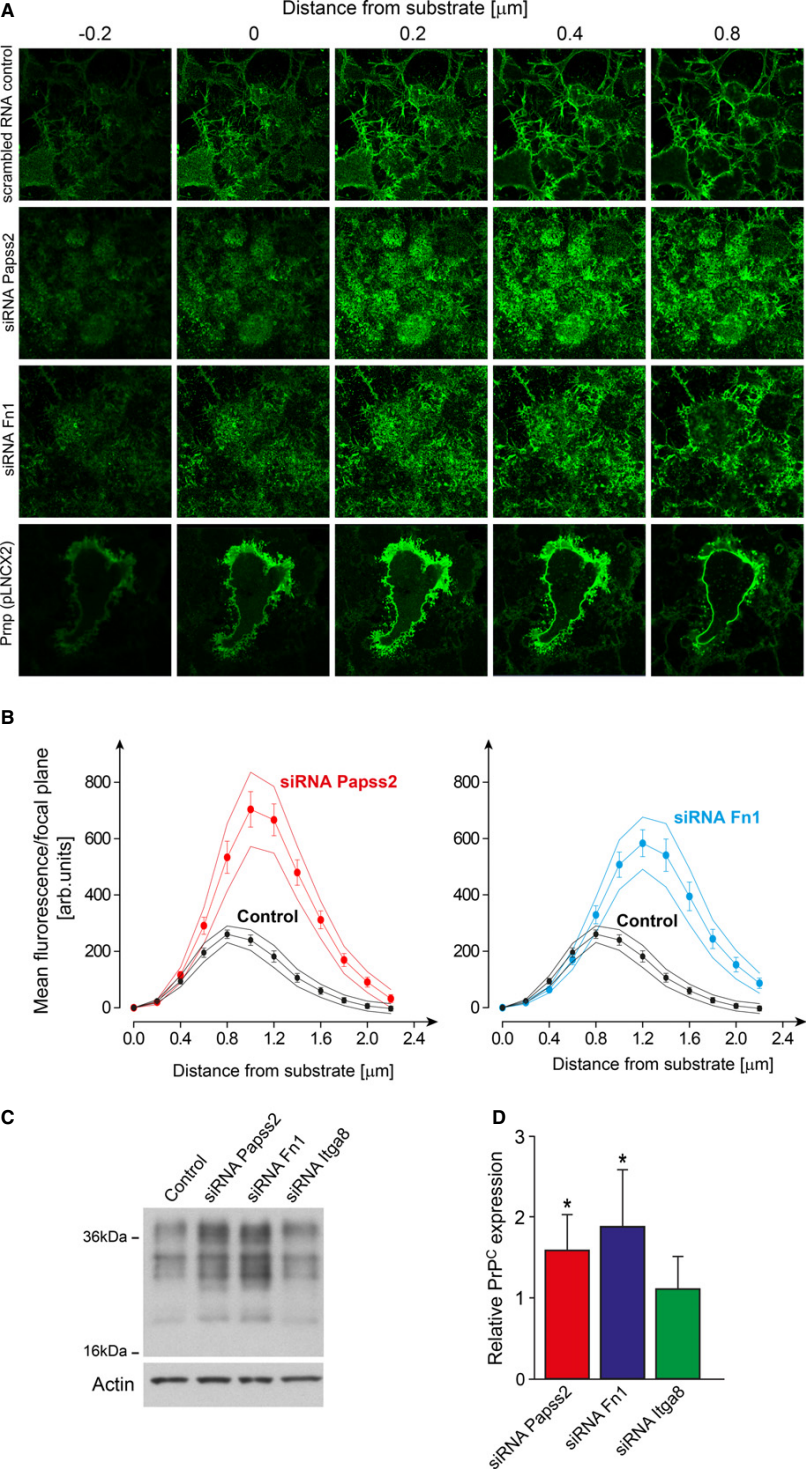
Phenotypic differences in PrP^C^ densities at the ECM upon *Papss2* and *Fn1* loss of function A Gene expression of *Papss2* and *Fn1* was silenced in R7 cells prior to immunolabelling with ICSM18. In addition, *Prnp* was overexpressed using pLNCX2 vector. Representative images of serial confocal z-stacks, acquired at identical laser setting, are shown. B Mean fluorescence intensities of *Papss2-* and *Fn1*-silenced cells labelled with ICSM18 and anti-mouse Alexa Fluor 488 antibodies were acquired according to the procedure described in Supplementary Fig S7. Mean fluorescence intensities of at least 40 intensity profiles ± standard error of the means, as well as upper and lower limits of 95% confidence intervals are shown. C, D PrP^C^ protein expression levels in R7 cells at 3 days after transfection with siRNA against *Papss2*,*Fn1, Itga8,* and scrambled RNA control are shown (C) and quantified for three biological repeats with β-actin as an endogenous control (D; anti-actin, clone ACTN05 (C4), Abcam). Significant changes (*P *< 0.05) between gene silencing of candidates and scrambled RNA control are shown (*).

Heparan sulphate mimetics are potent inhibitors of prion propagation (Schonberger *et al*, 2003), and heparin was suggested to displace PrP^C^ from lipid rafts (Taylor *et al*, 2009). We therefore examined whether *Papss2* knockdown is associated with phenotypic changes in PrP^C^ deposition in cells. Remarkably, *Papss2* as well as *Fn1* silencing markedly altered PrP^C^ distribution at the ECM (Fig9A). Serial scans along the z-axis in knockdown cells showed a higher granularity and fluorescence intensity of PrP^C^ at ECM, when compared to control (scrambled RNA) cells. In contrast, ectopic expression of *Prnp* (*Prnp* (pLNXC2)) led to increased fluorescence at plasma membrane, but not at the ECM level. To quantify these phenotypic alterations, we recorded serial z-stacks, determined the fluorescence intensity profiles of single cells, and computed the mean fluorescence intensities (Supplementary Fig S7A–C). When plotted against the distance from substrate, the mean fluorescence intensities of PrP^C^ in *Papss2*- and *Fn1*-silenced cells increased by more than 2-fold (Fig9B). A shift of the maximal intensity to larger distances from substrate in *Fn1*-silenced cells indicates that the amount of PrP^C^ deposited is increased. A significant increase in PrP^C^ expression levels in *Papss2*- and *Fn1*-silenced, but not in *Itga8*-silenced cells was confirmed on Western Blot (Fig9C and D).

## Discussion

We here present the first evidence for a gene regulatory network associated with susceptibility to prion propagation and modulated by the differentiation state of cells. Our data suggest that prion conversion is controlled by expression of genes with a role in the homeostasis of the ECM, a compartment characterised by abundant deposition of aberrant PrP^d^ in susceptible cells. Silencing of nine gene candidates expressed in prion-resistant revertants, *Fn1, Itga8, Chga, Iqgap2, Il11ra1, Micalcl, Papss2, Galt,* and *Rgs4*, significantly increased the rate of prion propagation. The RA-mediated downregulation of prion modifier genes led to a marked gain of susceptibility, suggesting that the rate of prion propagation is associated with transcriptional regulation of gene candidates. Loss of *Papss2* function led to undersulphation of HSPGs, increased deposition of PrP^C^ at the ECM, and a concomitant increase in prion conversion, indicating that HSPG sulphation is negatively correlated with susceptibility. Furthermore, inhibition of Fn1 binding to integrin α8 by the RGD peptide inhibited secretion of MMP2/9 and was associated with an increase in prion susceptibility. These data provide evidence that the ECM plays a critical role in the control of prion conversion.

Prion diseases are a group of fatal infectious neuronal disorders that are associated with the conversion of host-encoded PrP to misfolded pathogenic conformers and neurotoxicity (Prusiner & DeArmond, 1987). We here present the first *in vitro* evidence of an aberrant deposition of PrP^d^ at the ECM, a compartment that is deemed critical for prion replication (Caughey & Raymond, 1993b; Gabizon *et al*, 1993; Caughey *et al*, 1994). Delipidation with acetone and denaturation are critical to reveal PrP^d^ deposits at ECM and membrane level of cells, suggesting that in their aggregated state, the epitope is masked by lipids.

The ECM not only provides structural support for force transmission and tissue structure maintenance, but also plays a critical role in the regulation of physiological processes such as differentiation, migration, and intercellular communication. Transmembrane receptor proteins of the integrin family mechanically couple the actin cytoskeleton to the ECM by binding to Fn1 and other adhesion molecules, such as collagen and vitronectin. A convincing body of evidence suggests that MMP activation and expression is triggered by interaction of the cell adhesion molecule Fn1 or its proteolytic fragments with integrins (Esparza *et al*, 1999; Schedin *et al*, 2000; Yan *et al*, 2000; Thant *et al*, 2001; Forsyth *et al*, 2002; Loeser *et al*, 2003; Jin *et al*, 2011). This interaction can be inhibited by the RGD peptide (Koivunen *et al*, 1995), a tripeptide domain located in the 10th type-III module of Fn1 and site of cell attachment via β1 integrins. Integrin α8 regulates cell adhesion and migration by binding to Fn1, vitronectin, and tenascin-C in an RGD-dependent manner (Muller *et al*, 1995; Schnapp *et al*, 1995; Denda *et al*, 1998; Benoit *et al*, 2009). Remarkably, disruption of Fn1 binding to integrin α8 with the RGD peptide increased susceptibility and inhibited secretion of activated MMP2/9.

Whilst expression of nine prion modulators in revertants is negatively correlated with prion susceptibility, incubation of revertants with RA downregulated 18 gene candidates and led to a significant increase in prion propagation. In accord with this study, RA was shown, in human arterial smooth muscle cells, to inhibit the expression of Fn1 and MMPs, a phenotype that was associated with inhibition of migration and cell invasion (Axel *et al*, 2001; Scarpa *et al*, 2002).

A large body of evidence suggests that sulphated glycans and heparan sulphate mimetics act as potent inhibitors of prion propagation (Caughey & Race, 1992; Caughey *et al*, 1993a, 1994; Schonberger *et al*, 2003). Conversely, association of endogenous sulphated glycosaminoglycans (GAGs) with PrP^d^ deposits *in vivo* was taken as evidence that they may facilitate PrP^d^ formation (McBride *et al*, 1998). Inhibition of PAPS formation by two distinct approaches in this study, *Papss2* silencing and inhibition of sulfurylase, was associated with undersulphation of HSPGs and increased prion susceptibility. Our study does not support a direct interaction of sulphated HS chains with PrP^C^ *in vitro,* as shown with an anti-HS antibody, 10E4. In addition, no colocalisation between PrP^C^ and HSPGs was observed with the anti-HS antibody JM403. Heparin, a highly sulphated glycosaminoglycan was shown to displace PrP^C^ from rafts in a previous study and to promote its endocytosis (Taylor *et al*, 2009).

To address the question of how the sulphation status of HSPGs may affect prion propagation, we examined the distribution of PrP^C^ in *Papss2*-silenced R7 cells. Significantly more PrP^C^ was deposited in *Papss2* knockdown cells compared to controls, a phenotype that was also observed upon *Fn1* silencing. Notably, whilst ectopic expression of PrP^C^ failed to increase the rate of prion replication in revertants (Supplementary Table S1), silencing of *Papss2* and *Fn1* led to increased ECM deposition of PrP^C^ (Fig9) with a concomitant increase in conversion rates (Table1 and Supplementary Table S8). This implies that the subcellular deposition, rather than the relative levels of PrP^C^ expression, correlates with the corresponding prion conversion rates and may be due to a decrease in ECM proteolysis upon Fn1 downregulation ((Axel *et al*, 2001) and this study) and/or a disruption of FN matrix assembly by undersulphated HSPGs (Galante & Schwarzbauer, 2007). We therefore suggest that PrP^C^, deposited as a result of perturbed ECM homeostasis, may be a suitable substrate for prion conversion. A perturbation of matrix assembly has previously been reported in association with mutant *PAPSS2*. Chondrodysplasias, severe bone disorders, are associated with mutations in *PAPSS2* and *SLC26A2* and lead to reduced sulphate uptake, undersulphation of GAGs, and defective FN matrix assembly in cells (Ikeda *et al*, 2001; Galante & Schwarzbauer, 2007). Strikingly, a member of the Slc26a family of sulphate transporters, Slc26a4, is present in the gene signature of prion modifiers in our study, but its loss of function did not affect prion susceptibility, most likely due to the functional redundancy of this protein family, since Slc26a2, Slc26a4, Slc26a6, Slc26a8, and Slc26a11 are highly expressed in revertants.

A loss of ECM proteins in gamma-aminobutyric acid (GABA)-interneurons of Creutzfeldt-Jakob patients was suggested to precede extracellular prion deposition (Belichenko *et al*, 1999).

Other prion modifiers identified in this study are associated directly or indirectly to ECM homeostasis and remodelling. Chga, a member of the granin family of neuroendocrine secretory proteins, is located in secretory vesicles of neurons and neuroendocrine cells with a suggested role as a modulator of cell adhesion. Proteolytic processing of Chga to several bioactive peptides by plasmin (Metzboutigue *et al*, 1993; Colombo *et al*, 2002) and other proteases has been linked to modulation of cell adhesion (Metzboutigue *et al*, 1993) and negative regulation of angiogenesis (Belloni *et al*, 2007). Lrrn4, a transmembrane protein expressed in the hippocampus and cortex, contains leucine-rich repeat (LRR) motifs and fibronectin type-III-like repeats and is covalently linked to glycosaminoglycan side chains in its extracellular region (Bando *et al*, 2012). Lrrn4 was suggested to play an important role in hippocampus-dependent long-lasting memory (Bando *et al*, 2005). Il11ra1 is a type I cytokine receptor which contains two fibronectin type-III domains and one Ig-like C2-type (immunoglobulin-like) domain. Ablation of Il11ra1 is associated with defective decidualisation in the uterus of mice and leads to alterations in ECM components (White *et al*, 2004), but its role in ECM regulation is unknown.

In summary, we identified a gene regulatory network associated with prion replication and present evidence for the control of prion conversion at the ECM by an integrin-dependent activation of MMPs and the sulphation state of HSPGs. That genes involved in ECM homeostasis affect the kinetics of prion replication might help us to better understand the selective vulnerability of different neuronal populations during neurodegeneration (Guentchev *et al*, 1999; Siskova *et al*, 2013).

## Materials and Methods

### Antibodies

Mouse monoclonal anti-PrP (ICSM18) was obtained from D-Gen Limited (London, UK). Rabbit polyclonal anti-Fn1 (Cat# ab2413) was obtained from Abcam. Rabbit polyclonal anti-Lrrn4/Lrch4 (Cat# GTX1 12459) was purchased from GeneTex. Rabbit polyclonal antibody anti-IL11ra1 (Cat# 10264-1-AP) was purchased from Proteintech. Rabbit polyclonal anti-Itga8 (Cat# sc-25713) and rat monoclonal anti-NCAM (clone H28-123; Cat# sc-59934) were purchased from Santa Cruz. Rabbit polyclonal anti-CHGA (Cat# HPA017369) was obtained from Sigma. Mouse monoclonal anti-HS antibodies JM403 (Cat# 370730-1) and 10E4 (Cat# 370255-1) were obtained from Seikagaku (AMS Biotechnology, Abingdon OX14 4SE, UK). Mouse monoclonal anti-IQGAP2 (clone A2) was kindly provided by Prof. George S. Bloom. This antibody, a murine monoclonal IgG2a antibody against IQGAP2, was produced by hybridoma cells derived by fusion of Sp2/0-Ag14 myeloma cells with splenic lymphocytes isolated from a male A/J mouse that was immunised with purified, recombinant his-tagged human IQGAP2. The IQGAP2 was expressed in baculovirus-infected high five insect cells and purified by nickel affinity chromatography. The IQGAP2 coding sequence in the baculovirus was obtained by PCR amplification from a human brain cDNA library.

### Cell culture

N2a-derived cell lines were maintained in OptiMEM containing 10% foetal calf serum and 1% penicillin/streptomycin (OFCS). Cad5 cells were maintained in OptiMEM, supplemented with 10% bovine growth serum (HyClone) and 1% penicillin/streptomycin.

### Quantification of prion infection and rates of prion replication

Differences in the kinetics of cellular prion propagation were determined by quantifying the number of prion-infected cells during the course of three cell passages after infection using the Scrapie Cell Assay (SCA) (Klohn *et al*, 2003). The SCA is based on the microscopic detection of PK-resistant PrP (PrP^Sc^) in prion-permissive cells in an automated manner using spot detection software (Imaging Associates, UK) and Zeiss KS Elispot system. Cells were infected using serially diluted brain homogenates with known titres and the number of PrP^Sc^-positive cells expressed as Tissue Culture Infectious Units (TCIU). Briefly, 1.8 × 10^4^ cells were plated into wells of a 96-well plate. After 16 h, cells were incubated with 300 μl aliquots of serially diluted RML brain homogenate for 3 days. Cells were then split 1:8 for three subsequent passages, and aliquots of 2.5 × 10^4^ cells transferred onto Elispot plates (MultiScreen HTS-IP Filter Plate, Millipore). If not stated otherwise, the number of PrP^Sc^-positive cells was determined after digestion with 2.2 mU (0.5 μg) recombinant proteinase K (Roche Diagnostics) per millilitre of lysis buffer. The specific activity of PK (44 U/ml) was determined with the Chromozyme assay (Roche Diagnostics). Infectious titres were quantified from dilution series of RML brain homogenate I2424 (8.4 log LD50 units/g brain) and are expressed as tissue culture infectious units (TCIU) (Klohn *et al*, 2003). To determine the rate of prion replication, cells were infected with concentrated supernatants from chronically prion-infected cells. Due to the small size of these prion-infected nanoparticles, the inoculum does not have to be diluted out by serial cell passages as compared to the standard protocol (Klohn *et al*, 2003).

### RNA isolation and quality control

RNA from 8 × 10^6^ cells was isolated using RNeasy plus mini kit (Qiagen) with an average yield of 100 μg. To remove DNA contaminations, aliquots of RNA (10–30 μg) were incubated with 7 U RNase-free DNase I (Qiagen) in 50 μl RDD buffer at RT for 10 min. This step was repeated twice followed by heat inactivation of DNase at 70°C for 5 min. Subsequently, RNA was purified using RNeasy MinElute cleanup kit (Qiagen) according to the instructions of the manufacturer, and the RNA concentration was determined using a NanoDrop spectrophotometer (Labtech International). The purity of RNA samples was assessed using quantitative real-time PCR. Briefly, GAPDH expression levels of RNA isolates from cells were determined using one-step RT–PCR master mix (Applied Biosystems) with a rodent GAPDH probe (Applied Biosystems) in presence and absence of reverse transcriptase (RT). Differences between cycle threshold (Ct) values of purified RNA samples in presence and absence of RT were typically > 20, corresponding to a 40-fold difference between RNA over contaminating DNA concentration, confirming the quality of RNA isolates. Subsequently, cDNA was synthesised from 200 ng of total RNA from three biological replicates per cell line with cDNA archive kit (Applied Biosystems) according to the manufacturer's instructions.

### Microarray analysis

Relative gene expression levels were determined using GeneChip Mouse Genome 430 2.0 arrays (Affymetrix). Hybridisation, scanning, and microarray analysis were conducted at the Wolfson Institute for Biomedical Research and the UCL Cancer Institute. Microarrays were scanned using Genechip scanner 3000 and images analysed using GCOS version 1.2. Signal intensities were determined using one-step Tukey's biweight estimate with consideration of mismatch values to account for stray signal. Internal array controls were cross-checked for quality control. Log intensity ratios (M) versus average log intensity (A) were determined using R and Bioconductor and plotted before and after robust multi-array average (RMA)-normalisation. Box plots of array distributions are shown in Supplementary Fig S8. Pairwise significance analysis was performed using *t*-test with *P*-value cut-off of 0.01. The raw microarray data are deposited at NCBI Gene Expression Omnibus (GEO, accession number GSE56275).

### Quantitative real-time PCR

Relative gene expression levels were estimated by quantitative real-time PCR (qPCR) using 7500 Fast Real-Time PCR System (Applied Biosystems). Gene-specific dual-labelled probes (5′-FAM/3′-TAMRA) and primers were designed within the target sequence of the corresponding Affymetix probes using Primer Express software (Applied Biosystems) and synthesised by Eurofins MWG Operon (Ebersberg, Germany). Duplex PCR was carried out using TaqMan Gene Expression Master Mix (Applied Biosystems) in presence of VIC-conjugated mouse actin B (*Actb*) or *Gapdh* (Applied Biosystems). Cycling was at 50°C for 2 min, 95°C for 10 min, followed by 40–45 cycles of 95°C for 15 s, and 60°C for 1 min. Relative expression levels were calculated from serially diluted cDNA and were normalised to *Actb* or *Gapdh*.

### Overexpression of PrP^c^

To overexpress PrP^c^ in PK1 subclones, 1.2 × 10^5^ cells were plated per well of a 6-well plate and transfected with 2 μg of a murine Prnp expression vector or empty pLNCX2 vector, respectively, in presence of Lipofectamine 2000 (Invitrogen, Paisley, UK) according to the manufacturer's specifications. After 24 h, cells were expanded into 10-cm Petri dishes and selected the following day with 400 μg neomycin per ml OFCS. To exclude clonal effects on susceptibility levels, pools of antibiotic-resistant cells were used for experiments after 7–10 days of selection.

### Cloning shRNAs into pGIPZ and pRetroSuper

Short hairpin RNA (shRNA) constructs were expressed as human microRNA-30 (miR30) primary transcripts and contain a Drosha processing site. The hairpin stem is a 63-nucleotide (nt) stretch and consists of 22-nt sense dsRNA, a 19-nt loop (tagtgaagccacagatgta) from human miR30, followed by the 22-nt antisense dsRNA, and is flanked by miR30 flanking sequence on the 5′ (tgctgttgacagtgagcg) and 3′ (tgcctactgcctcgga) end of the stem. The design of shRNA was conducted with open-source algorithms from Thermo Scientific (siDESIGN Center tool) and the Hannon laboratory (shRNA retriever). 19-nt oligonucleotides were extended to 22nts using shRNA retriever. Constructs were amplified using Vent polymerase, the forward primer 5′-cagaaggctcgagaaggtatattgctgttgacagtgagcg-3′ and the reverse primer 5′-ctaaagtagccccttgaattccgaggcagtaggca-3′, containing XhoI and EcoRI restriction sites, respectively, according to the specification of the manufacturer Thermo Scientific. All 22-nt sense shRNA sequences used for gene silencing of target genes in this study are listed in Supplementary Table S12.

To silence *Prnp* expression in N_2_a cells a 19-mer, 5′-taggagatcttgactctga-3′, targeting the 3′ UTR of Prnp, was annealed and ligated into BglII and HinDIII sites of pRetroSuper vector and packaged into retroviral particles using Phoenix eco cells. Prion-susceptible PK1-10 cells were infected with viral supernatants in presence of 8 μg polybrene per ml OFCS, and antibiotic-resistant clones were selected with 4 μg puromycin/ml medium. Clone PK1-10/Si8kd (PK1 Prnp-RNAi) failed to propagate prions. Gene silencing by targeting of the 3′UTR region of Prnp enabled the reconstitution of PrP^c^ expression with a construct harbouring the Prnp open-reading frame.

### Generation of a Micalcl-YFP fusion protein

Micalcl (NM_027587) is poorly characterised, and Mammalian Gene Collection (MGC) clones are unavailable. We therefore assembled the full-length 2-kb coding sequence from synthetic gene fragments using gBlocks (Integrated DNA Technologies, IDT). Briefly, five gBlocks with a size ranging from 300 to 500 bp with flanking 20 bp complementary overhangs were designed and synthesised by IDT. Two and three gBlocks were assembled, respectively, using Gibson assembly master mix (New England Biolabs) according to the specification of the manufacturer. The product was amplified with forward primer 5′-atgaaccaaagagcaccatcgcctccaaagg-3′, reverse primer 5′-tcaagtcctgctgagctgacagcctctgg-3′ and AccuPrime Taq DNA polymerase high fidelity (Life Technologies). Micalcl was then cloned into the Gateway pCR8/GW/TOPO vector (Life Technologies) and transformed into TOP10 chemically competent E. coli and grown at 37°C overnight on agar plates, containing 100 μg spectinomycin per ml agar. Five colonies were picked and expanded, and plasmid DNA was isolated and purified using Qiagen spin miniprep kit. Plasmid-containing colonies were identified by EcoRI restriction digestion and sequenced to confirm the correct orientation of the insert. Micalcl was then transferred into a Gateway vector, pEYFP_C1 DEST, kindly provided by Prof. Stefan Wiemann (DKFZ, Germany) by recombinational cloning using LR Clonase II (Life Technologies).

### Gene silencing in cells and FACS enrichment

For gene silencing experiments, 1.1 × 10^6^ R7 cells were plated into 6-cm dishes and grown for 16 h. Five μg of pGIPZ vector, harbouring shRNA of the gene target, and 5 μl PLUS (Life Technologies) were added to 500 μl FBS-free OptiMEM and incubated for 5 min at RT. Seven microlitres of LTX (Life Technologies) were added, and the DNA-lipid mix incubated for 20 min. Cell medium was then replaced with DNA-lipid complex in a total of 3 ml OFCS. After 24 h, fluorescent cells were resuspended and enriched using a MoFlo (Beckman Coulter) according to the instructions of the manufacturer. Briefly, shRNA-expressing cells were enriched from a heterogeneous pool of fluorescent cells by gating GFP-positive cells in the 4th decade of the logarithmic fluorescence scale (see Fig3D). FACS-enriched cells were counted using a Coulter Counter Z2 (Beckman Coulter) at an upper threshold of 36 and a lower threshold of 12 and plated into wells of a 96-well plate at 1.8 × 10^4^ cells per well. After 16 h, cells were infected with 2 × 10^−5^ RML and processed according to the SCA protocol above.

Transient knockdown of gene candidates in cells without enrichment was conducted with double-stranded RNA dicer substrates (siRNAs) obtained from IDT. Briefly, 2 nmoles of siRNA was reconstituted in 200 μl duplex resuspension buffer (IDT). RNA-lipid complex formation was performed by adding 4 μl siRNA and 5.3 μl DharmaFect 3 (Thermo Scientific) to 100 μl FBS-free OptiMEM. After 20 min, RNA-lipid complexes were added to a total volume of 2 ml OFCS and combined with 2 ml of cell suspension at a concentration of 1 × 10^5^ cells per ml. Aliquots of 300 μl were then transferred out into 12 wells of a 96-well plate. After 2 days, cells were infected with RML and split the following day at a split ratio of 1:8. Cells were then processed according to the SCA protocol above.

### Detection of PrP^d^ deposits

Aliquots of 5 × 10^4^ chronically infected or uninfected N_2_a cells were plated into wells of 8-well chamber slides (Thermo Scientific) and cultured for three to four days. Cells were fixed with 4% formaldehyde/PBS for 12 min and washed once with PBS. Prolonged fixation greatly impedes PrP^d^ detection and has to be optimised for each cell line. To remove lipids, cells were incubated for 30 s with chilled acetone, methanol, or PBS and washed with PBS. Subsequently, cells were incubated with 3M guanidinium thiocyanate (Sigma) for 10 min and washed at least five times with PBS. Cells were then incubated with primary antibody in a 1:4 dilution of Superblock (Pierce) solution/PBS (v/v) for 1 h at RT or overnight at 4°C. Cells were rinsed with PBS twice and incubated with a 1:10,000 dilution of 4′,6-diamidino-2-phenylindole dihydrochloride (DAPI, 2 mg/ml DMSO) and Alexa Fluor-conjugated secondary antibodies (Life Technologies) at a dilution of 1:500 to 1:2,000 for 1 h. After two washes with PBS, cells were stored at 4°C until further processing. To exclude cross-reactivity of secondary antibodies during co-labelling experiments, batches of highly cross-adsorbed secondary antibodies were routinely tested using antibodies against distinct targets, that is, a rabbit-anti-EEA1 antibody (CST, #3288) or a rat anti-Lamp antibody (Santa Cruz, Cat# sc19992) and mouse anti-PrP antibody (ICSM18), followed by incubation with secondary antibodies. Immunofluorescence was analysed with a Zeiss LSM 710 confocal microscope and Zen imaging software (Carl Zeiss).

### Determination of PrP surface expression levels

Relative PrP surface expression levels of PK1 clones were determined by FACS. Briefly, 1 × 10^6^ cells were pelleted at 300 × g for 4 min and washed with PBS. Cell pellets were then fixed on ice with 4% paraformaldehyde/PBS for 30 min. After washing with PBS, cells were incubated with 5 μg of ICSM 18 in PBS/0.1% bovine serum albumin (BSA) for 30 min. Cells were washed again with PBS/0.1% BSA, spun, and incubated with a 1:200 dilution of Alexa Fluor 488-goat anti-mouse IgG (H+L) antibody (Invitrogen, Paisley, UK) in PBS/0.1% BSA for 30 min. After washing, PrP surface expression levels were determined using a FACS Calibur flow cytometer (BD Biosciences). Background levels of fluorescence were determined by labelling cells with secondary antibody only.

### Determination of cell population doubling times

Effects of RA treatment on cell doubling times of revertants and susceptible cells were determined using an automated cell counter, Z2 coulter counter (Beckman Coulter). In preliminary experiments, cell counting was compared to photometric determination with dyes WS1 and MTT. Owing to a high dynamic range of up to 3 orders of magnitude, cell doubling time was determined by automated cell counting. Briefly, cells were plated into 96-well plates at a concentration of 1.8 × 10^4^ cells per well and 16 h later incubated with various RA concentrations. Cells were harvested at 12, 24, 48, and 72 h after RA incubation by resuspending cell layers with multichannel pipettes and combined aliquots of 3 to 4 wells were counted.

### Analysis of confocal images

To collect confocal images of proteins expressed at the ECM, serial scans along the z-axis were conducted. A marked increase in fluorescence intensities at the level of the substrate delimits the ECM as depicted in Fig9. To quantify expression levels of candidate proteins, confocal images were collected at a 630-fold magnification (1.4 oil, Plan-Apochromat) and analysed using Volocity (Volocity, version 6.1.1). Where proteins were expressed at the ECM with no cell boundaries, data were analysed as follows. Nuclei were identified by DAPI in channel Ch-TS1 with an average size of 25 μm^3^. A dilation of 10 iterations from the nucleus was taken to represent the cell soma. Intensity values of candidate proteins, labelled with Alexa Fluor 488-conjugated secondary antibodies, were detected in channel ChS1-T2 on a single cell level and average intensities calculated with a cut-off of 5,000 units. The degree of colocalisation was assessed using Volocity by computing background-corrected threshold PCC values.

### PrP^C^ expression profiling at ECM

To assess the relative levels of PrP^C^ expressed at the ECM, cells were labelled with anti-PrP antibody ICSM18 and Alexa Fluor 488-conjugated secondary antibody and profiles of single cells from sequential z-stacks were analysed by Zen 2011 software (Zeiss, Cambridge, UK). Briefly, serial *z*-stacks of 0.2 μm were acquired for silenced and control cells at identical confocal settings. For image processing, fluorescence intensity profiles of single cells were acquired using Zeiss Zen software and incremental mean fluorescence intensities of sequential focal planes computed.

### MMP zymography

To determine activities of MMP2 and MMP9, 3 × 10^6^ cells were plated into 15-cm dishes in OFCS. After 16 h, cells were incubated with 1 μM RGD peptide (Santa Cruz Biotechnology) or vehicle (DMSO). After 72 h, supernatants were collected and cleared of cells and debris at 500 *g* for 10 min and 5,500 *g* for 20 min, respectively, using an Allegra 25R centrifuge (Beckman Coulter). Subsequently, supernatants were concentrated at 5,500 *g* on VivaSpin 20 columns (GE Healthcare Life Sciences) for 30 min. Concentrated samples were diluted 1:1 in Tris-glycine SDS gel loading buffer (Novex, Invitrogen) and separated by electrophoresis on 10% Tris-glycine gels containing 0.1% gelatine (Novex, Invitrogen) at 125 Volts for 90 min. Gels were incubated in 1× Zymogram renaturation and developing buffer (Novex, Invitrogen) according to the manufacturer's specification. Subsequently, gels were stained with SimplyBlue SafeStain (Novex, Invitrogen) for 6–12 h with 3–5 changes until protein bands were clearly visible.

### Statistical analysis

Statistical significance of differential gene expression was computed by non-parametric statistics using ‘Significance Analysis of Microarrays’ (SAM) (Tusher *et al*, 2001), and raw values corrected for multiple testing and expressed as false discovery rate (FDR) values (Benjamini & Hochberg, 1995). All other data are expressed as mean ± standard deviation (SD), unless otherwise stated. Comparisons of mean values were conducted by Student's *t*-test.
