# Supplementary material for: Identification of a gene regulatory network associated with prion replication
Source: EMBO J. 2014 May 19;33(14):1527–47. doi: 10.15252/embj.201387150 (PMC4198050; doi:10.15252/embj.201387150)
Supplement: Supplementary file 1 [file embj0033-1527-sd1.pdf]

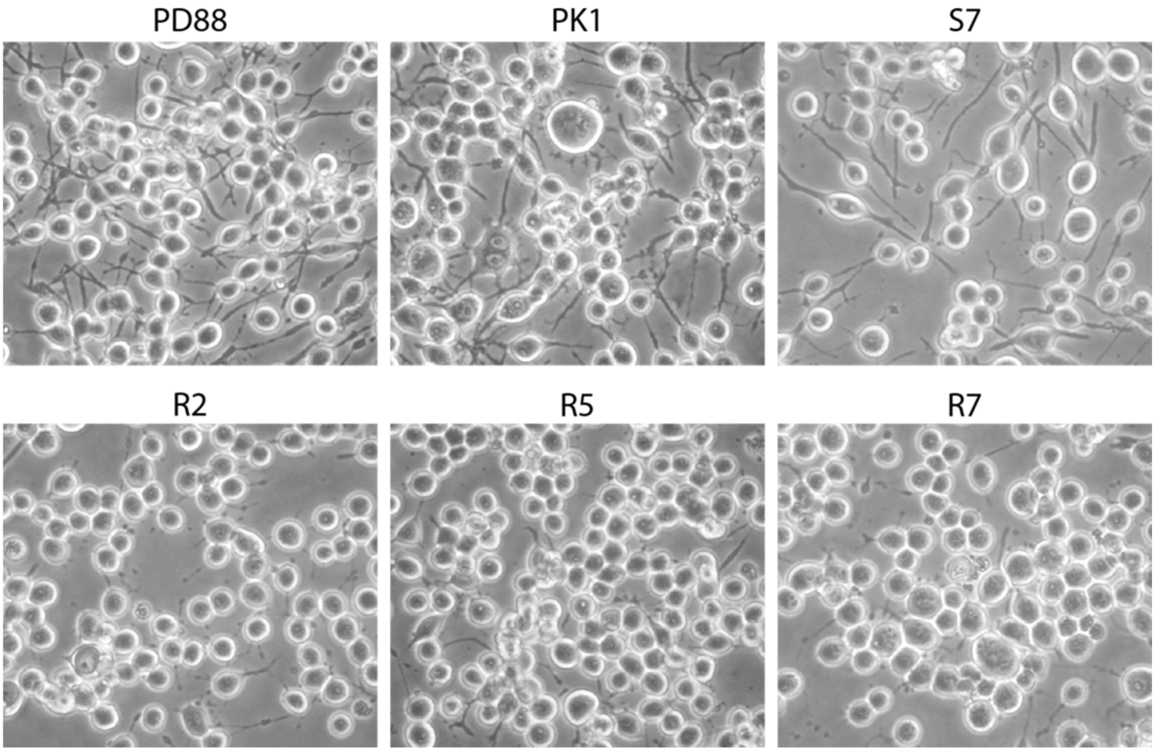

Figure S1 Susceptible cells are morphologically distinct from revertants and show a more differentiated phenotype. Bright field light microscopic images of revertant cell clones R2, R5 and R7 and susceptible sublines PD88, PK1 and S7 are shown. The morphology of susceptible cells is characterised by long neurites that generally form branches and networks with neurites from other cells. In contrast, revertants show a less differentiated phenotype with short mostly unbranched neurites.
