# Supplementary material for: Identification of a gene regulatory network associated with prion replication
Source: EMBO J. 2014 May 19;33(14):1527–47. doi: 10.15252/embj.201387150 (PMC4198050; doi:10.15252/embj.201387150)
Supplement: Supplementary file 2 [file embj0033-1527-sd2.pdf]

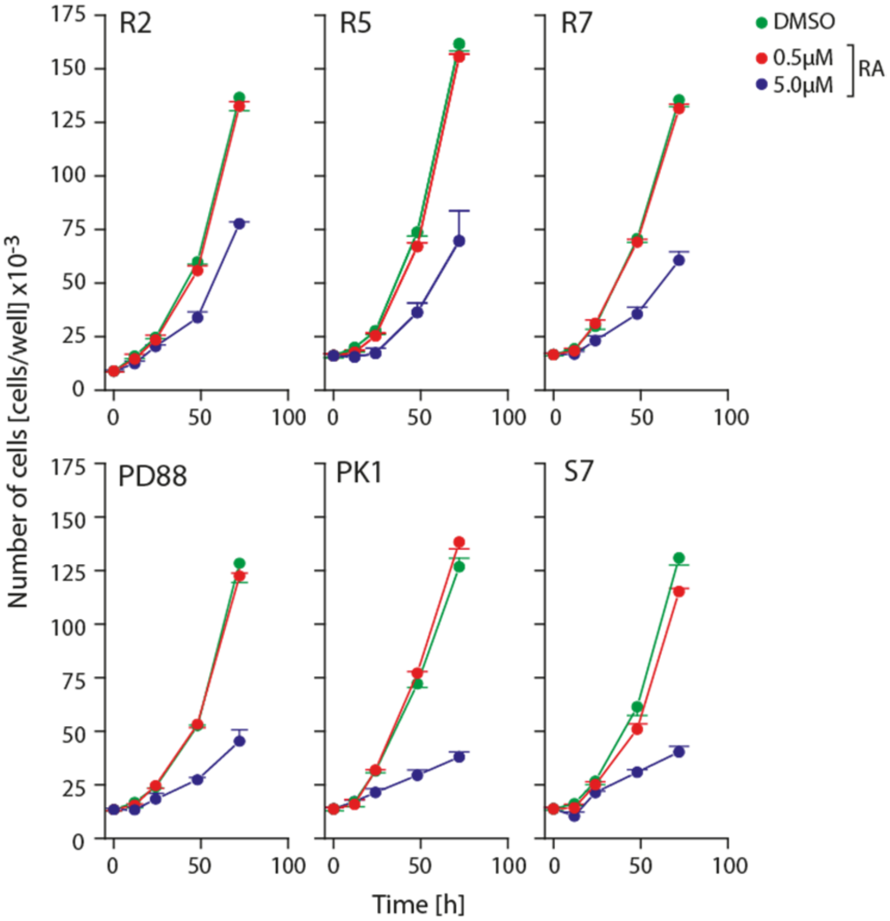

Figure S2 Low RA concentrations do not affect the growth rates of revertants. Eighteen thousand cells of the depicted cell lines were plated into wells of a 96-well plate containing 0.5 µM and 5 µM RA, respectively. Control cells were incubated with vehicle (DMSO). The number of cells was determined using a Coulter counter after 12h, 24h, 48h and 72h. Incubation of susceptible (PD88, S7) and revertant (R2, R5, R7) cells with 0.5 µM RA did not reduce the rate of cell doubling. The growth rates of all cell clones studied were reduced at a RA concentration of 5 µM.
