# Supplementary material for: Identification of a gene regulatory network associated with prion replication
Source: EMBO J. 2014 May 19;33(14):1527–47. doi: 10.15252/embj.201387150 (PMC4198050; doi:10.15252/embj.201387150)
Supplement: Supplementary file 3 [file embj0033-1527-sd3.pdf]

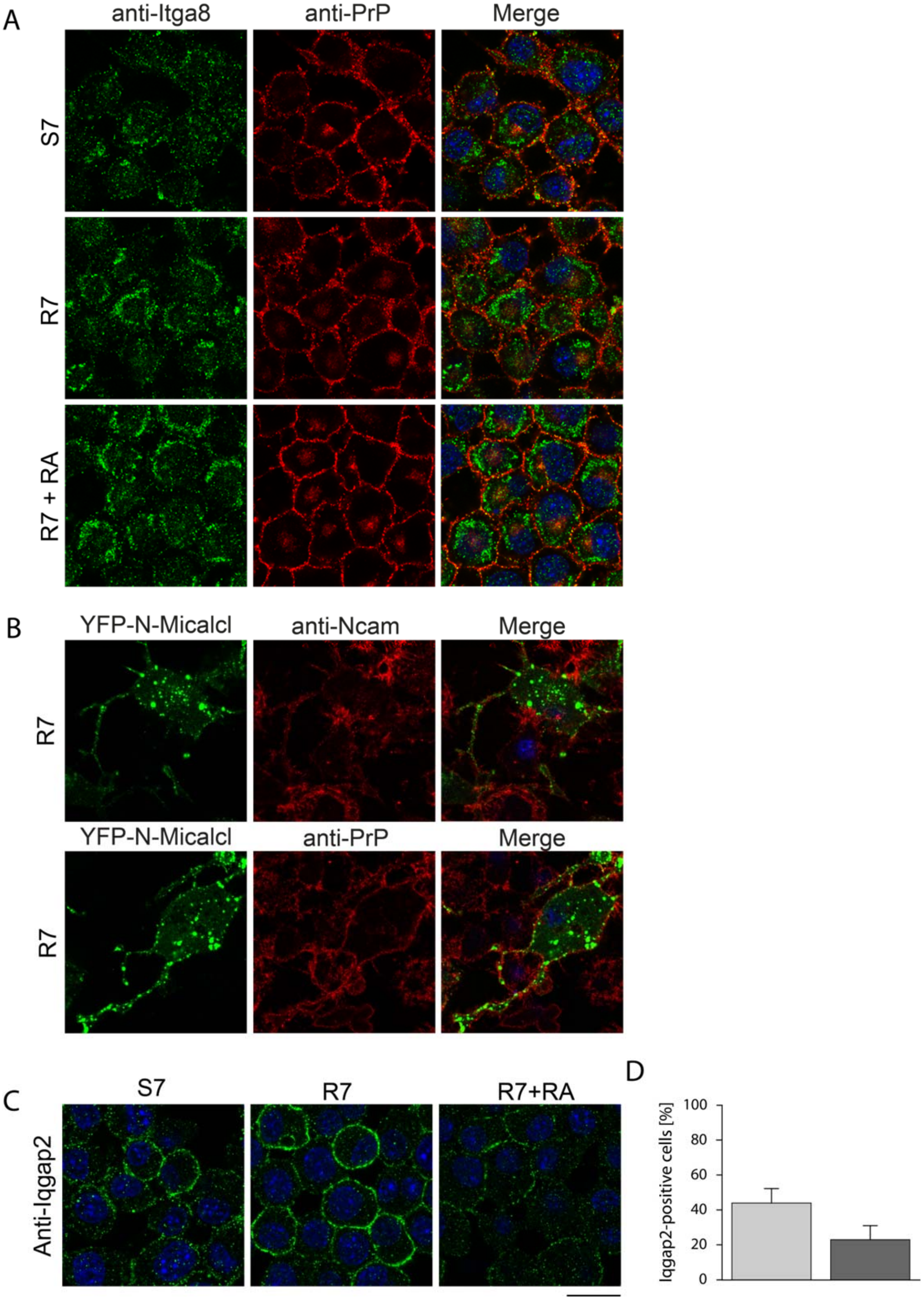

A
anti-Itga8
anti-PrP
Merge
S7
R7
R7 + RA
B
YFP-N-Micalcl
anti-Ncam
Merge
R7
YFP-N-Micalcl
anti-PrP
Merge
R7
C
S7
R7
R7+RA
Anti-Iqgap2
D
Iqgap2-positive cells [%]
100
80
60
40
20
0

Figure S3 Protein expression of integrin α8, Micalcl and Iqgap2 in revertant and susceptible cells. (A) Fixed and permeabilised R7 and S7 cells were co-labelled with a rabbit anti-integrin α8 antibody and mouse anti-PrP antibody ICSM18. In a parallel experiment R7 cells were incubated for 3 d with 0.5 μM RA (R7 + RA). (B) Micalcl was N-terminally fused with YFP and expressed in R7 cells. (C) S7 and R7 cells in presence and absence of RA were labelled with monoclonal anti-Iqgap2 antibody. (D) The number of Iqgap2-positive R7 (grey) and S7 (black) cells was analysed using Volocity analysis software (see Methods for details). Scale bar: 20 μm.
