# Supplementary material for: Identification of a gene regulatory network associated with prion replication
Source: EMBO J. 2014 May 19;33(14):1527–47. doi: 10.15252/embj.201387150 (PMC4198050; doi:10.15252/embj.201387150)
Supplement: Supplementary file 4 [file embj0033-1527-sd4.pdf]

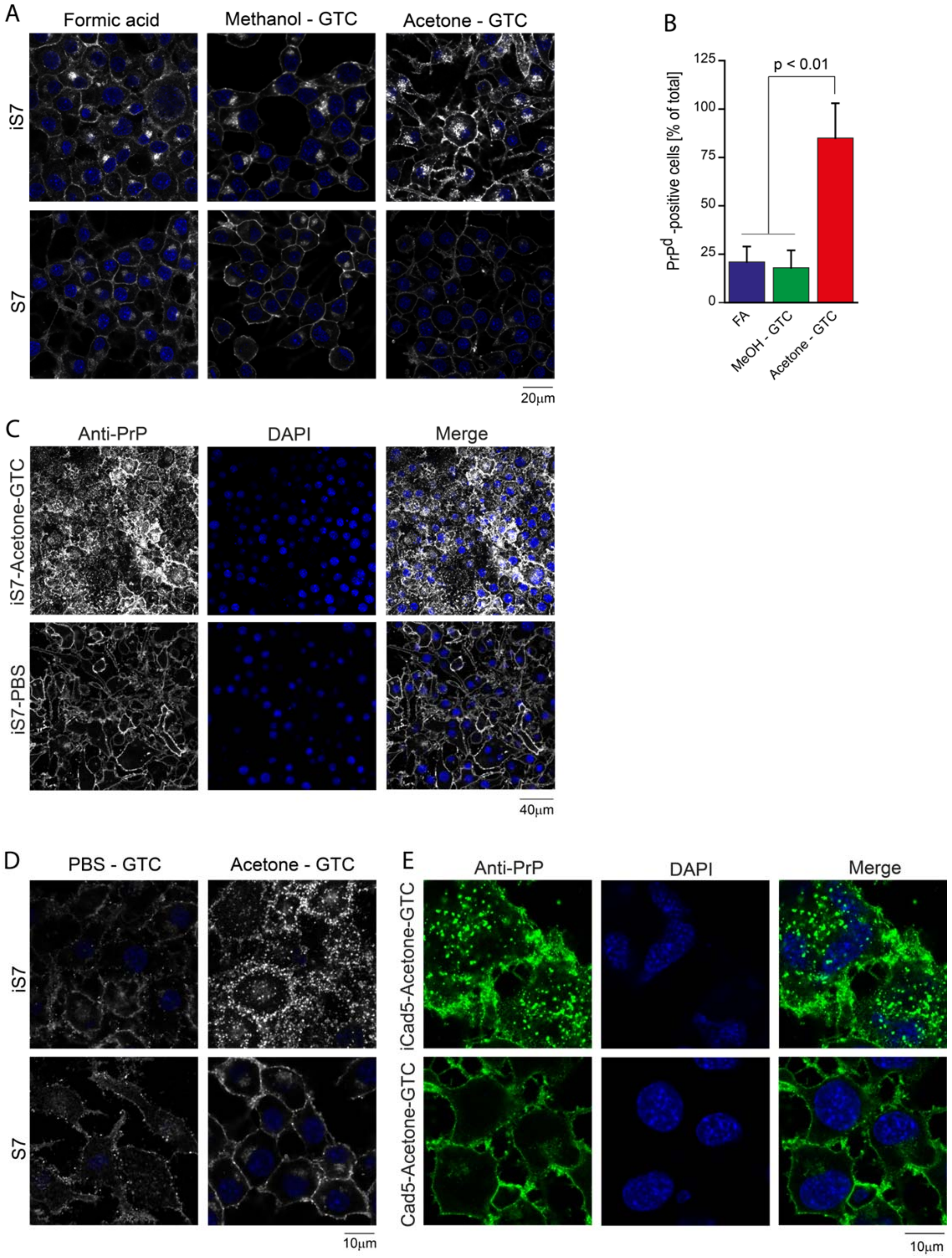

Figure S4 Distinct subcellular location and abundance of PrP^d deposits detected by formic acid, methanol and acetone pretreatment. (A) Chronically infected (iS7) and uninfected (S7) cells

were plated into chamber slides and fixed with paraformaldehyde. Cells were then treated with formic acid or delipidated with methanol or acetone as described in Supplemental Experimental Procedures. Delipidated cells were incubated with 3 M GTC for ten minutes and washed five times with PBS. Deposits of PrP^d were detected after immunolabelling cells with anti-PrP antibody ICSM18. (B) The number of cells with PrP^d deposits was determined in 20 frames and represents mean values ± SD. (C) Low magnification images show PrP^d deposits at the ECM level of iS7 cells after treatment with acetone/GTC, but not with PBS. (D) Aberrant PrP^d deposits at the plasma membrane level are shown at cross-sections above the ECM. (E) Aberrant PrP^d deposits in chronically prion-infected Cad5 (iCad5) cells after delipidation with acetone. ICad5 and uninfected Cad5 cells were fixed with paraformaldehyde, delipidated with acetone and denatured with GTC. Representative images are shown.
