# Supplementary material for: Identification of a gene regulatory network associated with prion replication
Source: EMBO J. 2014 May 19;33(14):1527–47. doi: 10.15252/embj.201387150 (PMC4198050; doi:10.15252/embj.201387150)
Supplement: Supplementary file 5 [file embj0033-1527-sd5.pdf]

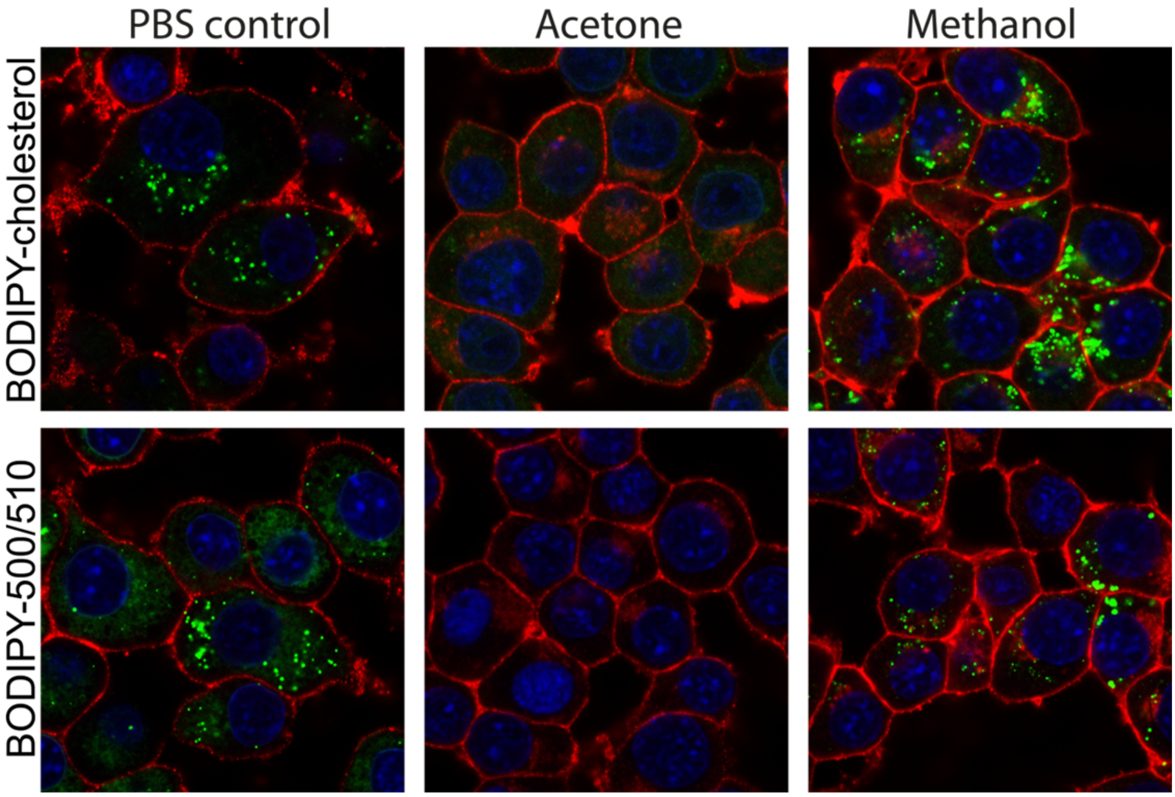

Figure S5 Treatment of fixed cells with acetone removes neutral lipids and cholesterol. S7 cells, labelled overnight with BODIPY-500/510 and BODIPY-cholesterol were fixed with PFA for 12 minutes and incubated with chilled acetone, methanol and PBS for 30 seconds. Cells were subsequently labelled with ICSM18, anti-mouse Alexa Fluor 568 and DAPI and confocal images acquired.
