# Supplementary material for: Identification of a gene regulatory network associated with prion replication
Source: EMBO J. 2014 May 19;33(14):1527–47. doi: 10.15252/embj.201387150 (PMC4198050; doi:10.15252/embj.201387150)
Supplement: Supplementary file 6 [file embj0033-1527-sd6.pdf]

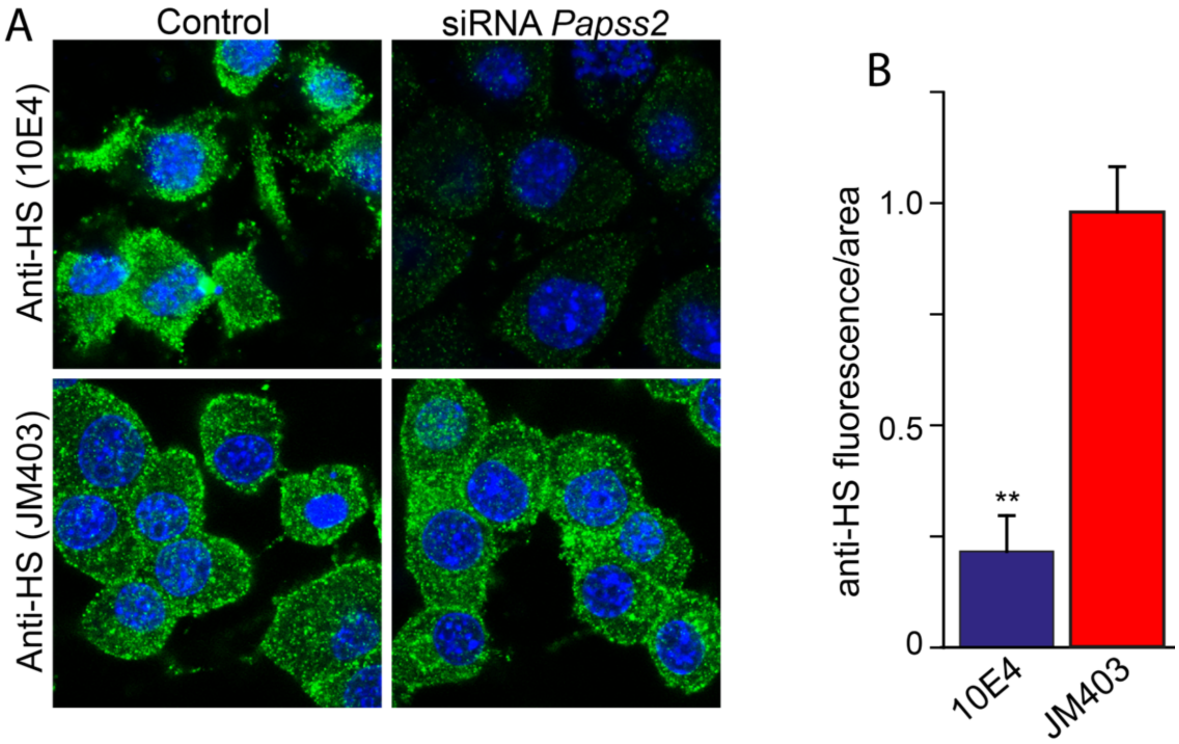

Figure S6 Binding of anti-HS antibody 10E4, but not of JM403 is dependent on HS sulfation. (A) R2 cells were transfected with siRNA against *Papss2* and scrambled RNA control. After 3 days cells were labelled with anti-HS antibodies 10E4 and JM403 and fluorescence intensities were recorded. (B) Quantitative analysis of fluorescence intensities using Volocity, $p < 0.001$ (**).
