# Supplementary material for: Identification of a gene regulatory network associated with prion replication
Source: EMBO J. 2014 May 19;33(14):1527–47. doi: 10.15252/embj.201387150 (PMC4198050; doi:10.15252/embj.201387150)
Supplement: Supplementary file 7 [file embj0033-1527-sd7.pdf]

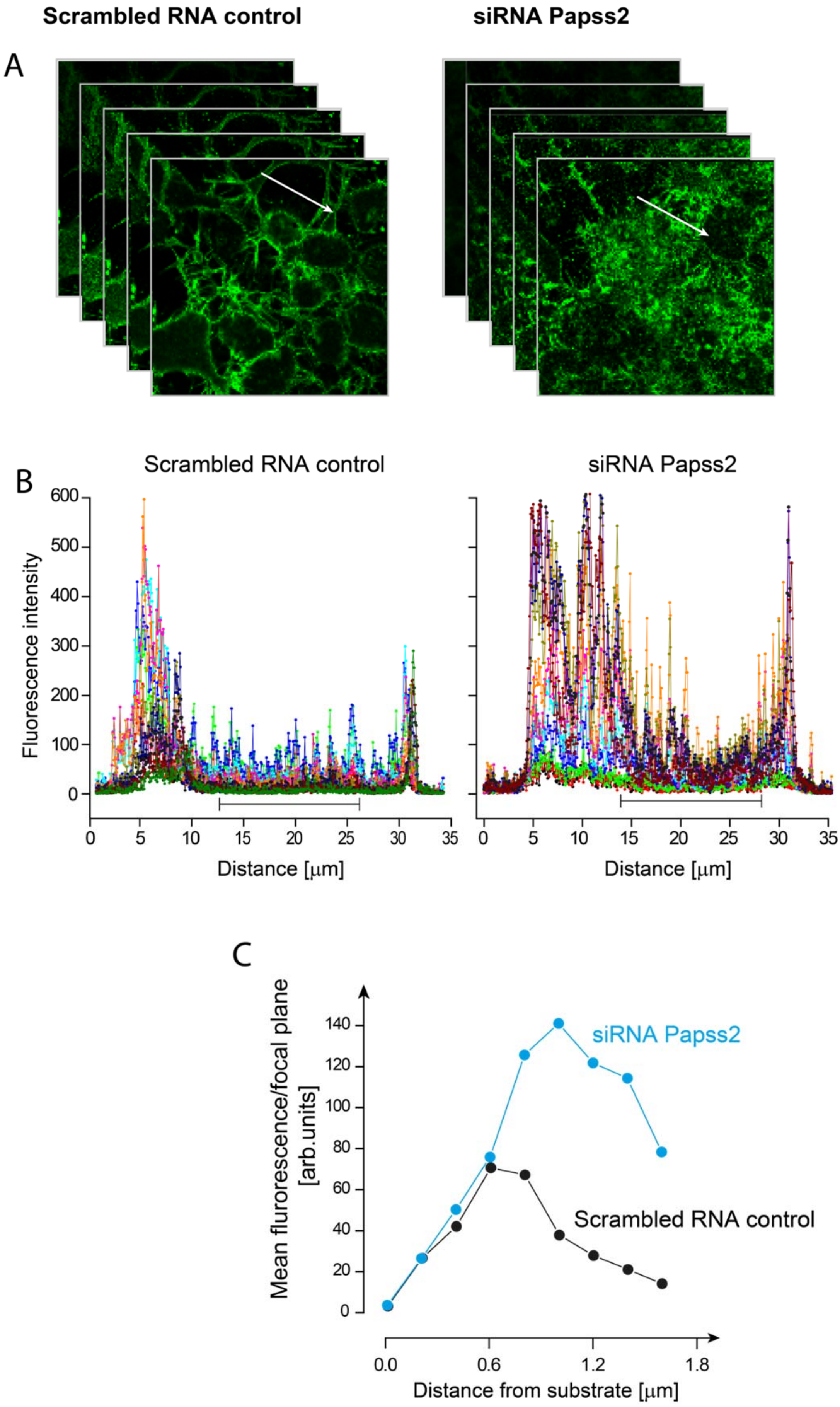

Scrambled RNA control
siRNA Papss2
A
B
Scrambled RNA control
siRNA Papss2
Fluorescence intensity
600
500
400
300
200
100
0
0
5
10
15
20
25
30
35
Distance [μm]
C
Mean flurorescence/focal plane [arb.units]
140
120
100
80
60
40
20
0
siRNA Papss2
Scrambled RNA control
0.0
0.6
1.2
1.8
Distance from substrate [μm]

Figure S7 ECM intensity profiling of PrP^C labelled cells. (A) *Papss2*-silenced and control R7 cells were labelled with ICSM18 and anti-mouse Alexa Fluor 488 conjugated secondary antibody. Serial z-stacks of 0.2 microns were acquired for *Papss2* silenced and control cells under identical confocal settings. For image processing fluorescence intensity profiles of single cells (arrow) were acquired using Zeiss Zen software. (B) Superimposed fluorescence intensity profiles of twelve focal planes are shown. To determine mean fluorescence intensities of sequential focal planes, lines of 12 µm length (scale bars) within the plasma membrane boundaries of cells were selected. (C) Mean fluorescence intensities of sequential focal planes are plotted against the distance from substrate.
