# Supplementary material for: Identification of a gene regulatory network associated with prion replication
Source: EMBO J. 2014 May 19;33(14):1527–47. doi: 10.15252/embj.201387150 (PMC4198050; doi:10.15252/embj.201387150)
Supplement: Supplementary file 8 [file embj0033-1527-sd8.pdf]

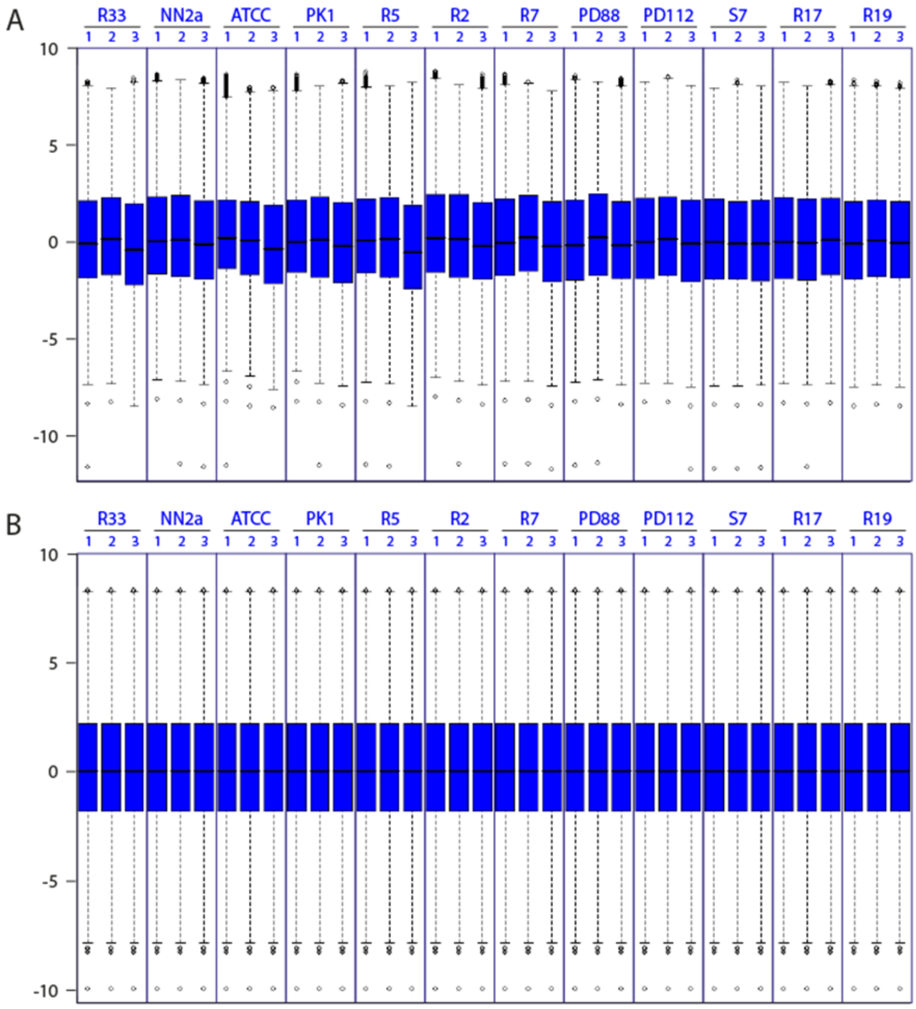

Figure S8 Quality control and normalisation of microarray data. Box plots representing the difference between microarray expression chips before (A) and after (B) normalisation using Robust Microarray Averaging (RMA) (Bolstad et al., 2003;Irizarry et al., 2003). Boxes show the 25th and 75th percentiles in the distribution of log2-transformed intensities. RMA normalisation resulted in low inter-array variability as shown by the consistency of median values and similar intensity distributions.

Bolstad BM, Irizarry RA, Astrand M, and Speed TP (2003) A comparison of normalization methods for high density oligonucleotide array data based on variance and bias. *Bioinformatics,* **19,** 185-193.

Irizarry RA, Hobbs B, Collin F, Beazer-Barclay YD, Antonellis KJ, Scherf U, and Speed TP (2003) Exploration, normalization, and summaries of high density oligonucleotide array probe level data. *Biostatistics,* **4,** 249-264.
