# Supplementary material for: Identification of a gene regulatory network associated with prion replication
Source: EMBO J. 2014 May 19;33(14):1527–47. doi: 10.15252/embj.201387150 (PMC4198050; doi:10.15252/embj.201387150)
Supplement: Supplementary file 9 [file embj0033-1527-sd9.pdf]

Clone	Susceptibility	PrP^{Sc+} cells [TCIU/ml]* Mean ± Stdv		Relative PrP^{c} expression# Fluorescence intensities	
		endogenous	elevated PrP^{c}	endogenous	elevated PrP^{c}
PK1$_{Prnp\text{-}kd}$	res	0 ± 0	396 ± 39	58	142
R2	rev	77 ± 11	57 ± 12	163	262
R5	rev	15 ± 6	12 ± 6	111	145
R7	rev	31 ± 8	21 ± 5	144	224
PK1	sus	526 ± 38	640 ± 23	165	211
PD88	sus	666 ± 77	679 ± 72	158	204
PD112	sus	172 ± 29	201 ± 46	135	252

* The number of PrP^{Sc}-positive cells after prion infection of distinct cell clones at endogenous and elevated levels of PrP, expressed as TCIU/ml was determined as described in Methods.

# Relative PrP expression levels of cells were determined using FITC-conjugated anti-PrP antibodies.

Supplementary Table S1: PrP overexpression does not render prion-revertant cells susceptible. Relative levels of susceptibility and PrP surface expression of revertant (rev) and susceptible (sus) PK1 clones expressing endogenous or elevated levels of PrP^{c} were determined three passages after incubation with a 10^{-5} dilution of RML. Infectious titres, expressed as TCIU/ml were determined at a proteinase K concentration of 8.8 mU/ml lysis buffer as described in Methods. To control that expression of *Prnp* allotype A (*Prnp*^{a}) confers susceptibility to prion propagation we reconstituted stably *Prnp*-silenced PK1 cells (PK1$_{Prnp\text{-}kd}$) with *Prnp*^{a}.
