# Supplementary material for: Identification of a gene regulatory network associated with prion replication
Source: EMBO J. 2014 May 19;33(14):1527–47. doi: 10.15252/embj.201387150 (PMC4198050; doi:10.15252/embj.201387150)
Supplement: Supplementary file 10 [file embj0033-1527-sd10.pdf]

a: Genes expressed in prion-susceptible cells

Gene name	Description	FC
G0s2	G0/G1 switch gene 2	-7.8
Actg2	actin, gamma 2, smooth muscle, enteric	-6.1
Afp	alpha fetoprotein	-5.3
Cth	cystathionase (cystathionine gamma-lyase)	-5.2
P2rx3	purinergic receptor P2X, ligand-gated ion channel, 3	-4.9
Csprs	component of Sp100-rs	-4.3
Calcrl	calcitonin receptor-like	-4.2
Taf7l	TAF7-like RNA polymerase II, TATA box binding protein (TBP)-associated factor	-4.2
Mamdc2	MAM domain containing 2	-3.9
Bex 1	brain expressed gene 1	-3.7
Pla2g2e	phospholipase A2, group IIE	-3.2
Ifitm3	interferon induced transmembrane protein 3	-3.2
Slamf9	SLAM family member 9	-3.2
Hopx	homeobox only domain	-2.9
Cald1	caldesmon 1	-2.7
Dgat2	diacylglycerol O-acyltransferase 2	-2.6
Abcb1a	ATP-binding cassette, sub-family B (MDR/TAP), member 1A	-2.6
Fam149a	family with sequence similarity 149, member A	-2.5
Tmsb4x	thymosin, beta 4, X chromosome	-2.5
Tnfaip2	tumor necrosis factor, alpha-induced protein 2	-2.5
Tshz1	teashirt zinc finger family member 1	-2.5
Gpr85	G protein-coupled receptor 85	-2.4
Nckap1l	NCK associated protein 1 like	-2.4
Ptger4	prostaglandin E receptor 4 (subtype EP4)	-2.3
Fry	furry homolog (Drosophila)	-2.3
Atf3	activating transcription factor 3	-2.3
Ywhae	tyrosine 3-monooxygenase/tryptophan 5-monooxygenase activation protein,epsilon polypeptide	-2.2
Tmtc4	transmembrane and tetratricopeptide repeat containing 4	-2.2
Car6	carbonic anhydrase 6	-2.2
Pdgfrb	platelet derived growth factor receptor, beta polypeptide	-2.1
Bhlhb2	basic helix-loop-helix domain containing, class B2	-2.1
Zdbf2	zinc finger, DBF-type containing 2	-2.1
Dis3	mitotic control homolog (S. cerevisiae)	-2.0
Ranbp5	RAN binding protein 5	-2.0
Adm	adrenomedullin	-2.0
Gadd45a	growth arrest and DNA-damage-inducible 45 alpha	-2.0
Xmr	Xlr-related, meiosis regulated	-2.0
Rpl22l1	ribosomal protein L22 like 1	-2.0
Pcbp2	poly(rC) binding protein 2	-2.0
Plekha1	pleckstrin homology domain containing, family A (phosphoinositide binding specific) member 1	-2.0
Dock2	dedicator of cyto-kinesis 2	-2.0
Gpt2	glutamic pyruvate transaminase (alanine aminotransferase) 2	-2.0
Pdlim7	PDZ and LIM domain 7	-1.9
Ret	ret proto-oncogene	-1.9
Prepl	prolyl endopeptidase-like	-1.9
Cyp51	cytochrome P450, family 51	-1.9
Cacng3	calcium channel, voltage-dependent, gamma subunit 3	-1.9
Ptbp2	polypyrimidine tract binding protein 2	-1.9
Tpm2	tropomyosin 2, beta	-1.9
Akr1b7	aldo-keto reductase family 1, member B7	-1.9

Syt4	synaptotagmin IV	-1.9
Dp1	deleted in polyposis 1	-1.9
Vps54	vacuolar protein sorting 54 (yeast)	-1.9
Scn3b	sodium channel, voltage-gated, type III, beta	-1.9
Phf20l1	PHD finger protein 20-like 1	-1.9
C730049O14Rik	RIKEN cDNA C730049O14 gene	-1.8
Slc2a1	solute carrier family 2 (facilitated glucose transporter), member 1	-1.8
Ddr2	discoidin domain receptor family, member 2	-1.8
Csrp1	cysteine and glycine-rich protein 1	-1.8
Txndc10	thioredoxin domain containing 10	-1.8
3110043O21Rik	RIKEN cDNA 3110043O21 gene	-1.8
Paqr3	progestin and adipoQ receptor family member III	-1.8
Atrnl1	attractin like 1	-1.8
Diap3	diaphanous homolog 3 (Drosophila)	-1.8
Stom	stomatin	-1.8
Stk17b	serine/threonine kinase 17b (apoptosis-inducing)	-1.8
Cnn2	calponin 2	-1.8
Rapgef5	Rap guanine nucleotide exchange factor (GEF) 5	-1.8
Rbm26	RNA binding motif protein 26	-1.7
Dock9	dedicator of cytokinesis 9	-1.7
Myadm	myeloid-associated differentiation marker	-1.7
Slc6a15	solute carrier family 6 (neurotransmitter transporter), member 15	-1.7
Iigp2	interferon inducible GTPase 2	-1.7
Tmeff2	transmembrane protein with EGF-like and two follistatin-like domains 2	-1.7
Stard5	StAR-related lipid transfer (START) domain containing 5	-1.7
1700112E06Rik	RIKEN cDNA 1700112E06 gene	-1.7
Mib1	mindbomb homolog 1 (Drosophila)	-1.7
4930503L19Rik	RIKEN cDNA 4930503L19 gene	-1.7
Anxa5	annexin A5	-1.7
Cry1	cryptochrome 1 (photolyase-like)	-1.7
Phr1	pam, highwire, rpm 1	-1.7
Slain1	Slain motif family, member 1	-1.7
Prune2	prune homolog 2 (Drosophila)	-1.7
Mef2b	myocyte enhancer factor 2B	-1.7
Mfge8	milk fat globule-EGF factor 8 protein	-1.7
Tigd2	tigger transposable element derived 2	-1.7
Capn2	calpain 2	-1.7
Klf5	Kruppel-like factor 5	-1.7
Btg1	B-cell translocation gene 1, anti-proliferative	-1.7
Adcy7	adenylate cyclase 7	-1.7
Egr3	early growth response 3	-1.7
Dach2	dachshund 2 (Drosophila)	-1.7
Cln5	ceroid-lipofuscinosis, neuronal 5	-1.6
Rnf138	ring finger protein 138	-1.6
Tcte3	t-complex-associated testis expressed 3	-1.6
Gm337	gene model 337, (NCBI)	-1.6
Tnfsf13b	tumor necrosis factor (ligand) superfamily, member 13b	-1.6
Pygl	liver glycogen phosphorylase	-1.6
Txnl1	thioredoxin-like 1	-1.6
Sc4mol	sterol-C4-methyl oxidase-like	-1.6
Stk24	serine/threonine kinase 24 (STE20 homolog, yeast)	-1.6
Dcbld1	discoidin, CUB and LCCL domain containing 1	-1.6
Hmgcll1	3-hydroxymethyl-3-methylglutaryl-Coenzyme A lyase-like 1	-1.6
Cpe	carboxypeptidase E	-1.6

b: Genes expressed in prion-resistant revertant cells

Gene name	Description	FC
Klf3	Kruppel-like factor 3 (basic)	4.3
Id4	inhibitor of DNA binding 4	3.4
Slc26a4	solute carrier family 26, member 4	3.0
Gm1683	predicted gene 3716	3.0
Hist3h2a	histone 3, H2a	3.0
Lect1	leukocyte cell derived chemotaxin 1	2.9
Fst	follistatin	2.8
Sgk1	serum/glucocorticoid regulated kinase 1	2.7
Rgs4	regulator of G-protein signaling 4	2.6
Igfbp7	insulin-like growth factor binding protein 7	2.5
Micalcl	MICAL C-terminal like	2.5
AI428936	expressed sequence AI428936	2.5
Tesc	tescalcin	2.4
Hist3h2ba	histone 3, H2ba	2.3
Igsf5	immunoglobulin superfamily, member 5	2.3
Il11ra1	interleukin 11 receptor, alpha chain 1	2.3
Galt	galactose-1-phosphate uridyl transferase	2.3
Lor	loricrin	2.3
Papss2	3'-phosphoadenosine 5'-phosphosulfate synthase 2	2.2
Gng4	guanine nucleotide binding protein (G protein), gamma 4 subunit	2.2
Bambi	BMP and activin membrane-bound inhibitor	2.1
Asah2	N-acylsphingosine amidohydrolase 2	2.1
Slc40a1	solute carrier family 40 (iron-regulated transporter), member 1	2.1
Mkx	homeobox protein mohawk	2.1
Chga	chromogranin A	2.1
Gnas	guanine nucleotide-binding protein G(s) subunit alpha isoforms short	2.1
Rnd2	Rho family GTPase 2	2.1
Ptgr1	prostaglandin reductase 1	2.0
Hint2	histidine triad nucleotide binding protein 2	2.0
Dlc1	deleted in liver cancer 1	2.0
Cenph	centromere autoantigen H	2.0
Dmkn	dermokine	2.0
Gm5918	predicted gene 5918	2.0
Iqgap2	IQ motif containing GTPase activating protein 2	2.0
Lrrn4	leucine rich repeat neuronal 4	2.0
Ptplad2	protein tyrosine phosphatase-like A domain containing 2	1.9
Procr	protein C receptor, endothelial	1.9
Scg2	secretogranin II	1.8
Chrna3	cholinergic receptor, nicotinic, alpha polypeptide 3	1.8
Flywch2	FLYWCH family member 2	1.8
Itga8	integrin alpha 8	1.8
Vegfc	vascular endothelial growth factor C	1.8
Parp8	poly (ADP-ribose) polymerase family, member 8	1.8
Pik3ip1	phosphoinositide-3-kinase interacting protein 1	1.8
Usp18	ubiquitin specific peptidase 18	1.7
Fn1	fibronectin 1	1.7
Ech1	enoyl coenzyme A hydratase 1, peroxisomal	1.7
Rsph9	radial spoke head 9 homolog (Chlamydomonas)	1.7
6330403K07Rik	RIKEN cDNA 6330403K07 gene	1.7
Glrx1	glutaredoxin 1 (thioltransferase)	1.7
Fastkd3	FAST kinase domains 3	1.7
Nfe2l2	nuclear factor, erythroid derived 2, like 2	1.7
Nfkbia	nuclear factor of kappa light chain gene enhancer in B-cells inhibitor, alpha	1.7
Mblac2	metallo-beta-lactamase domain containing 2	1.7
AI429214	expressed sequence AI429214	1.7
Mras	muscle and microspikes RAS	1.7

Hist1h4j	histone 1, H4j	1.7
Rasl11a	RAS-like, family 11, member A	1.7
Zfp131	zinc finger protein 131	1.7
Akr1c12	aldo-keto reductase family 1, member C12	1.7
C77080	expressed sequence C77080	1.7
Rfesd	Rieske (Fe-S) domain containing	1.7
Ccdc106	coiled-coil domain containing 106	1.7
Mrpl36	mitochondrial ribosomal protein L36	1.7
Serf1	small EDRK-rich factor 1	1.7
Slc36a1	solute carrier family 36 (proton/amino acid symporter), member 1	1.7
Pomc1	pro-opiomelanocortin-alpha	1.7
Gtf2h2	general transcription factor II H, polypeptide 2	1.7
Zfp595	zinc finger protein 595	1.7
Htra4	HtrA serine peptidase 4	1.7
Cdk7	cyclin-dependent kinase 7	1.7
Ssbp2	single-stranded DNA binding protein 2	1.6
Spata11	spermatogenesis associated 11	1.6
Klf6	Kruppel-like factor 6	1.6
Sirt7	sirtuin 7	1.6
6330512M04Rik	RIKEN cDNA 6330512M04 gene	1.6
Slc30a1	solute carrier family 30 (zinc transporter), member 1	1.6
Sccpdh	saccharopine dehydrogenase (putative)	1.6
Parp14	poly (ADP-ribose) polymerase family, member 14	1.6
Cetn3	centrin 3	1.6
Abhd3	abhydrolase domain containing 3	1.6
Solt	SoxLZ/Sox6 leucine zipper binding protein in testis	1.6
Frmd6	FERM domain containing 6	1.6
Sesn3	sestrin 3	1.6
Sdf2	stromal cell derived factor 2	1.6
Unc5c	unc-5 homolog C (C. elegans)	1.6

Supplementary Table S2: Genes expressed in susceptible (a) and prion-resistant revertant (b) cells are listed according to their fold change (FC) between revertant and susceptible cells. Normalised expression values were corrected for multiple testing at high stringency with a false-discovery rate (FDR) < 0.01. FC values of genes expressed in susceptible and revertant cells are denoted negative and positive, respectively.
