# Supplementary material for: Identification of a gene regulatory network associated with prion replication
Source: EMBO J. 2014 May 19;33(14):1527–47. doi: 10.15252/embj.201387150 (PMC4198050; doi:10.15252/embj.201387150)
Supplement: Supplementary file 11 [file embj0033-1527-sd11.pdf]

GOTERM: Development (16 genes)
Enrichment score: 2.6, p-value: 1.9 x 10^{-3}; FDR: 3.5

IL15	Interleukin 15
ACTG2	Actin, gamma 2, smooth muscle, enteric
WWTR1	WW domain containing transcription regulator 1
PAPSS2	3'-Phosphoadenosine 5'-phosphosulfate synthase 2
ID4	Inhibitor of DNA binding 4
FST	Follistatin
IGFBP7	Follistatin-like 2
SLC26A4	Solute carrier family 26, member 4
LECT1	Leukocyte cell derived chemotaxin 1
SOX9	Sry-box containing gene 9
S100A6	S100 Calcium binding protein A6 (calcyclin)
PTGER4	Prostaglandin E receptor 4 (subtype EP4)
DCAMKL1	Double cortin and calcium/calmodulin-dependent protein kinase-like 1
TGFB2	Transforming growth factor, beta 2
AFP	Alpha fetoprotein
DOCK2	Dedicator of cytokinesis

GOTERM: Cell differentiation (18 genes)
Enrichment score: 2.6, p-value: 1.7 x 10^{-3}; FDR: 4.1

IL15	Interleukin 15
WWTR1	WW domain containing transcription regulator 1
ID4	Inhibitor of DNA binding 4
FST	Follistatin
DCAMKL1	Double cortin and calcium/calmodulin-dependent protein kinase-like 1
TGFB2	Transforming growth factor, beta 2
SOX9	Sry-box containing gene 9
S100A6	S100 calcium binding protein A6 (calcyclin)
LECT1	Leukocyte cell derived chemotaxin 1
DOCK2	Dedicator of cytokinesis 2
TNFAIP2	Tumor necrosis factor, alpha-induced protein 2
MIB1	Mindbomb homology 1 (drosophila)
VEGFC	vascular endothelial growth factor c
Ret	Ret proto-oncogene
IL11RA1	Interleukin 11 receptor, alpha chain 1
LOR	Loricrin
PDLIM7	Pdz and lim domain 7
NFKBIA	Nuclear factor of kappa light chain gene enhancer in b-cells inhibitor, alpha

GOTERM: Negative regulation of cell differentiation (5 genes)
Enrichment score: 2.7, p-value: 7.2 x 10^{-3}; FDR: 2.5

NFKBIA	Nuclear factor of kappa light chain gene enhancer in b-cells inhibitor, alpha
ID4	Inhibitor of DNA binding 4
FST	Follistatin
WWTR1	WW domain containing transcription regulator 1
MIB1	Mindbomb homology 1 (drosophila)

Supplementary Table S3: Functional annotation clustering of genes differentially expressed between prion-resistant revertant and susceptible clones. Functional annotation clustering was conducted using Database of Annotation, Visualisation and Integrated Discovery (DAVID) (Huang et al., 2009).

Huang DW, Sherman BT, and Lempicki RA (2009) Systematic and integrative analysis of large gene lists using DAVID bioinformatics resources. *Nature Protocols,* **4,** 44-57.
