# Supplementary material for: Identification of a gene regulatory network associated with prion replication
Source: EMBO J. 2014 May 19;33(14):1527–47. doi: 10.15252/embj.201387150 (PMC4198050; doi:10.15252/embj.201387150)
Supplement: Supplementary file 12 [file embj0033-1527-sd12.pdf]

Treatment		Prion susceptibility [TCIU/ml]				
**RA**	**RML**	**R2**	**R5**	**R7**	**PD88**	**PK1**
**-**	**+**	14 ± 12	12 ± 6	72 ± 14	385 ± 148	613 ± 105
**+**	**+**	375 ± 170	96 ± 66	552 ± 266	758 ± 139	122 ± 92
**+**	**-**	7 ± 3	20 ± 11	9 ± 6	15 ± 7	7 ± 3
**-**	**-**	7 ± 4	6 ± 2	9 ± 7	8 ± 3	15 ± 12

Supplementary Table S4: A retinoic acid-induced phenotypic switch from prion-resistant revertants to susceptible cells. Eighteen thousand revertant (R2, R5 and R7) and susceptible cells (PD88 and PK1) were cultured for 16 h and incubated with 0.5 μM RA or vehicle (DMSO) for three days. Cells were split at a ratio of 1:8 and 24 h later challenged with RML mouse prions at a dilution of 10^{-5} (2.2 x 10^3 i.c. LD_{50}/ml). Prion susceptibility of cells, expressed as TCIU/ml was determined by SCA as specified in Methods.
