# Supplementary material for: Identification of a gene regulatory network associated with prion replication
Source: EMBO J. 2014 May 19;33(14):1527–47. doi: 10.15252/embj.201387150 (PMC4198050; doi:10.15252/embj.201387150)
Supplement: Supplementary file 13 [file embj0033-1527-sd13.pdf]

(**a**) Genes downregulated in R7 cells upon RA treatment

Gene name	Description	FC
Igfbp5	insulin-like growth factor binding protein 5	-11.2
**Id4**	**inhibitor of DNA binding 4**	**-5.1**
Pdlim3	PDZ and LIM domain 3	-4.7
BC024760	cDNA sequence BC024760	-4.0
Lix1	limb expression 1 homolog (chicken)	-3.4
Myb	myeloblastosis oncogene	-3.3
Nrp2	neuropilin 2	-3.3
**Fst**	**follistatin**	**-3.2**
**Igsf5**	**immunoglobulin superfamily, member 5**	**-2.9**
Htr3a	5-hydroxytryptamine (serotonin) receptor 3A	-2.9
Cpeb2	cytoplasmic polyadenylation element binding protein 2	-2.9
**Fn1**	**fibronectin 1**	**-2.8**
Zcchc12	zinc finger, CCHC domain containing 12	-2.6
Cxxc4	CXXC finger 4	-2.5
**Lrch4**	**leucine-rich repeats and calponin homology domain containing 4**	**-2.5**
Myl9	myosin, light polypeptide 9, regulatory	-2.5 **Rgs4**
	**regulator of G-protein signaling 4**	**-2.5**
Tox	thymocyte selection-associated high mobility group box	-2.5
Tle1	transducin-like enhancer of split 1, homolog of Drosophila E(spl)	-2.4
Msrb3	methionine sulfoxide reductase B3	-2.4
Rragd	Ras-related GTP binding D	-2.4
Socs2	suppressor of cytokine signaling 2	-2.4
Igfbp4	insulin-like growth factor binding protein 4	-2.3
**Iqgap2**	**IQ motif containing GTPase activating protein 2**	**-2.3 Micalcl MICAL**
**C-terminal like**	**-2.3**	
Gpr22	G protein-coupled receptor 22	-2.2
**Slc26a4**	**solute carrier family 26, member 4**	**-2.2**
Hs3st3b1	heparan sulfate (glucosamine) 3-O-sulfotransferase 3B1	-2.1
Dscaml1	Down syndrome cell adhesion molecule-like 1	-2.1
Tle4	transducin-like enhancer of split 4, homolog of Drosophila E(spl)	-2.0
Gsn	gelsolin	-2.0 **Dlc1 deleted in liver**
**cancer 1**	**-2.0**	
Tmod1	tropomodulin 1	-2.0
Galnt3	UDP-N-acetyl-alpha-D-galactosamine:polypeptide N-acetylgalactosaminyltransferase 3	-2.0
F13a1	coagulation factor XIII, A1 subunit	-2.0
Gchfr	GTP cyclohydrolase I feedback regulator	-2.0
**Galt**	**galactosyltransferase**	**-2.0**
**Bambi**	**BMP and activin membrane-bound inhibitor, homolog** (Xenopus laevis)	**-2.0**
Klf6	Kruppel-like factor 6	-2.0
:	:	:
**Il11ra1**	**interleukin 11 receptor, alpha chain 1**	**-2.0**
**Itga8**	**integrin alpha 8**	**-2.0**
**Papss2**	**3'-phosphoadenosine 5'-phosphosulfate synthase 2**	**-2.0**

(**b**) Genes upregulated in R7 cells upon RA treatment

Gene name	Description	FC
Cyp26b1	cytochrome P450, family 26, subfamily b, polypeptide 1	+63.2
Csnk	casein kappa	+61.0
Ifi203	interferon activated gene 203	+12.7
Mmp15	matrix metallopeptidase 15	+10.7
Matn2	matrilin 2	+5.4
**Nckap1l**	**NCK associated protein 1 like**	**+4.7**
Slc25a20	solute carrier family 25, member 20	+4.6
Ccnjl	cyclin J-like	+4.3
Ptp4a3	protein tyrosine phosphatase 4a3	+4.2
Hadh2	hydroxyacyl-Coenzyme A dehydrogenase type II	+3.7

Ramp1	receptor (calcitonin) activity modifying protein 1	+3.7
Arhgap28	Rho GTPase activating protein 28	+3.6
Elf5	E74-like factor 5	+3.5
Lrrfip1	leucine rich repeat (in FLII) interacting protein 1	+3.5
Plekhg1	pleckstrin homology domain containing, family G member 1	+3.5
Irs1	insulin receptor substrate 1	+3.5
Mela	melanoma antigen	+3.4
Bpil2	bactericidal/permeability-increasing protein-like 2	+3.3
Gdpd5	glycerophosphodiester phosphodiesterase domain containing 5	+3.3
Sgpp2	sphingosine-1-phosphate phosphotase 2	+3.3
Abca1	ATP-binding cassette, sub-family A (ABC1), member 1	+3.0
AI839735	expressed sequence AI839735	+3.0
Eml5	echinoderm microtubule associated protein like 5	+2.9
Sprr1a	small proline-rich protein 1A	+2.8
Meis1	myeloid ecotropic viral integration site 1	+2.7
Ttc8	tetratricopeptide repeat domain 8	+2.7
D9Ertd280e	DNA segment, Chr 9, ERATO Doi 280, expressed	+2.6
Rps6	ribosomal protein S6	+2.6
Ugp2	UDP-glucose pyrophosphorylase 2	+2.6
Dlgh2	discs, large homolog 2 (Drosophila)	+2.6
Usp43	ubiquitin specific peptidase 43	+2.5
Ctsb	cathepsin B	+2.3
Nrip1	nuclear receptor interacting protein 1	+2.3
Gas1	growth arrest specific 1	+2.3
Ches1	checkpoint supressor 1	+2.1
4930404N11Rik	RIKEN cDNA 4930404N11 gene	+2.1
Cd9	CD9 antigen	+2.1
St6gal1	ST6 beta-galactosamide alpha-2,6-sialyltranferase 1	+2.0
Rps15a	ribosomal protein S15a	+2.0
6230424C14Rik	RIKEN cDNA 6230424C14 gene	+2.0
Agps	alkylglycerone phosphate synthase	+2.0
Smug1	single-strand selective monofunctional uracil DNA glycosylase	+2.0
Adam19	a disintegrin and metallopeptidase domain 19 (meltrin beta)	+2.0
Ppfibp2	PTPRF interacting protein, binding protein 2 (liprin beta 2)	+2.0
Arhgef3	Rho guanine nucleotide exchange factor (GEF) 3	+2.0
Isx	intestine specific homeobox	+2.0
Usp43	ubiquitin specific peptidase 43	+2.0
Erc2	ELKS/RAB6-interacting/CAST family member 2	+2.0
Dlgh2	discs, large homolog 2 (Drosophila)	+2.0
Eml5	echinoderm microtubule associated protein like 5	+2.0
**Tshz1**	**teashirt zinc finger family member 1**	**+2.0**
Rasl10b	RAS-like, family 10, member B	+2.0
Chm	choroidermia	+2.0

Supplementary Table S5: Differential gene expression in revertant R7 cells at 6 hours after RA treatment. Genes downregulated (a) and upregulated (b) upon RA incubation are shown. Genes are listed according to their fold change (FC). Normalised expression values were corrected for multiple testing at high stringency with a FDR < 0.01. FC values of genes downregulated and upregulated upon RA treatment are denoted negative and positive, respectively. In bold are genes that are also found in the list of differentially expressed genes between susceptible and revertant cells (Supplementary Table S2).
