# Supplementary material for: Identification of a gene regulatory network associated with prion replication
Source: EMBO J. 2014 May 19;33(14):1527–47. doi: 10.15252/embj.201387150 (PMC4198050; doi:10.15252/embj.201387150)
Supplement: Supplementary file 14 [file embj0033-1527-sd14.pdf]

Abbreviation shRNA conctruct	*Rel. rate of prion propagation*			*Gene silencing*	
	FC	*SD*	*t-test*	*% kd*	*SD*
shRNA-Galt.1	1.60	0.32	2.2×10^{-3}	42	20
shRNA-Galt.2	0.91	0.28	3.9×10^{-1}	22	21
shRNA-Galt.3	1.18	0.20	1.7×10^{-1}	14	11
shRNA-Galt.4	1.82	0.50	2.5×10^{-5}	41	17
shRNA-Galt.5	0.45	0.26	4.2×10^{-3}	64	13
shRNA-Galt.6	0.88	0.20	2.9×10^{-2}	28	26
shRNA-Iqgap2.1	1.29	0.14	3.8×10^{-2}	24	19
shRNA-Iqgap2.2	1.44	0.36	1.2×10^{-2}	33	9
shRNA-Iqgap2.3	0.99	0.26	9.5×10^{-1}	23	8
shRNA-Iqgap2.4	5.65	1.07	1.3×10^{-17}	55	27
shRNA-Iqgap2.5	0.92	0.26	3.0×10^{-1}	38	18
shRNA-Iqgap2.6	0.81	0.24	2.1×10^{-1}	32	9
shRNA-Slc26a4.1	1.03	0.38	8.3×10^{-1}	88	13
shRNA-Slc26a4.2	1.25	0.58	1.5×10^{-1}	69	34
shRNA-Slc26a4.3	1.48	0.48	1.9×10^{-2}	83	13
shRNA-Slc26a4.4	0.93	0.55	6.7×10^{-1}	78	14
shRNA-Slc26a4.5	1.23	0.71	3.4×10^{-1}	89	23
shRNA-Chga.1	4.41	1.36	9.8×10^{-7}	58	12
shRNA-Chga.2	4.30	0.80	4.3×10^{-8}	55	21
shRNA-Chga.3	1.12	0.43	5.2×10^{-1}	32	25
shRNA-Chga.4	1.30	0.63	8.2×10^{-1}	23	7
shRNA-Chga.5	1.14	0.57	6.5×10^{-1}	18	24
shRNA-Id4.1	0.87	0.27	7.9×10^{-2}	46	4
shRNA-Id4.2	1.46	0.37	3.8×10^{-2}	43	4
shRNA-Id4.3	1.12	0.39	3.7×10^{-1}	39	2
shRNA-Id4.4	0.75	0.31	1.2×10^{-2}	72	8
shRNA-Id4.5	0.80	0.44	2.0×10^{-1}	51	14
shRNA-Id4.7	0.95	0.20	5.8×10^{-1}	46	19
shRNA-Id4.8	0.88	0.22	2.8×10^{-1}	48	31
shRNA-IL11ra1.1	2.46	0.92	5.6×10^{-5}	54	8
shRNA-IL11ra1.2	3.15	0.79	2.6×10^{-7}	84	18
shRNA-IL11ra1.3	2.24	0.98	3.4×10^{-3}	50	30
shRNA-IL11ra1.4	0.93	0.26	5.2×10^{-1}	12	9
shRNA-IL11ra1.5	1.14	0.32	7.2×10^{-1}	31	8
shRNA-IL11ra1.6	1.09	0.49	8.7×10^{-1}	19	12
shRNA-Lrrn4.1	1.03	0.53	9.0×10^{-1}	73	13
shRNA-Lrrn4.2	0.75	0.27	2.7×10^{-2}	77	17
shRNA-Lrrn4.3	0.54	0.12	7.9×10^{-3}	81	16
shRNA-Lrrn4.4	0.68	0.18	1.1×10^{-1}	85	4
shRNA-Fn1.1	1.81	0.45	5.6×10^{-5}	72	12
shRNA-Fn1.2	3.15	0.66	9.9×10^{-5}	89	17
shRNA-Fn1.3	1.74	0.44	8.6×10^{-4}	70	14
shRNA-Fn1.5	1.53	0.83	5.4×10^{-1}	61	13

shRNA-Fn1.6	3.39	0.42	3.5 x 10^{-10}	87	23
shRNA-Micalcl.1	2.85	0.59	5.9 x 10^{-9}	95	15
shRNA-Micalcl.2	1.89	0.37	2.8 x 10^{-4}	87	33
shRNA-Micalcl.3	2.47	0.16	9.2 x 10^{-6}	83	16
shRNA-Micalcl.4	1.66	0.33	1.7 x 10^{-2}	86	4
shRNA-Rgs4.1	2.54	0.49	4.3 x 10^{-7}	75	19
shRNA-Rgs4.2	2.61	0.42	8.3 x 10^{-4}	69	12
shRNA-Rgs4.3	2.01	0.42	5.7 x 10^{-5}	77	14
shRNA-Rgs4.4	1.90	0.43	7.6 x 10^{-4}	55	22
shRNA-Rgs4.5	3.44	0.99	1.5 x 10^{-7}	80	16
shRNA-Rgs4.6	1.82	0.31	7.6 x 10^{-4}	61	3
shRNA-Rgs4.7	2.91	0.27	1.7 x 10^{-8}	65	17
shRNA-Papss2.1	2.32	0.13	1.6 x 10^{-9}	48	9
shRNA-Papss2.2	2.36	0.30	4.7 x 10^{-9}	70	17
shRNA-Papss2.3	1.23	0.43	2.6 x 10^{-1}	21	13
shRNA-Papss2.4	1.23	0.16	7.5 x 10^{-2}	16	15
shRNA-Igsf5.1	0.87	0.33	4.3 x 10^{-1}	36	4
shRNA-Igsf5.2	1.42	0.41	6.5 x 10^{-2}	2	1
shRNA-Igsf5.3	1.13	0.63	5.3 x 10^{-1}	34	4
shRNA-Igsf5.4	0.94	0.28	7.5 x 10^{-1}	68	5
shRNA-Bambi.1	0.92	0.31	5.6 x 10^{-1}	75	6
shRNA-Bambi.2	1.19	0.23	1.4 x 10^{-1}	65	10
shRNA-Fst.1	0.97	0.39	8.0 x 10^{-1}	24	6
shRNA-Fst.3	1.15	0.08	1.6 x 10^{-1}	28	14
shRNA-Fst.4	0.69	0.36	2.6 x 10^{-2}	41	20
shRNA-Fst.5	0.98	0.09	8.1 x 10^{-1}	13	6
shRNA-Fst.6	1.07	0.34	5.4 x 10^{-1}	81	8
shRNA-Dlc1.1	0.73	0.13	4.9 x 10^{-2}	41	12
shRNA-Dlc1.2	1.34	0.27	2.0 x 10^{-2}	73	4
shRNA-Dlc1.3	1.03	0.08	5.0 x 10^{-1}	61	17
shRNA-Itga8.1	2.70	0.12	2.8 x 10^{-9}	85	13
shRNA-Itga8.2	0.89	0.26	5.0 x 10^{-1}	49	21

Supplementary Table S6: Validation of susceptibility gene candidates by gene silencing. Revertant R7 cells transiently expressing shRNA against distinct gene candidates using the bicistronic vector pGIPZ were enriched for highly GFP-fluorescent cells and subsequently infected with RML mouse prions at a dilution of 2 x 10^{-5}. The rates of prion propagation were determined by SCA and normalized against cells transfected with non-silencing control vectors (NSC GIPZ). Relative rates of prion propagation expressed as fold change (FC) to controls (NSC) ± SD for at least three independent experiments are shown. The level of gene knockdown (% kd) was determined as described in Methods.
