# Supplementary material for: Identification of a gene regulatory network associated with prion replication
Source: EMBO J. 2014 May 19;33(14):1527–47. doi: 10.15252/embj.201387150 (PMC4198050; doi:10.15252/embj.201387150)
Supplement: Supplementary file 15 [file embj0033-1527-sd15.pdf]

Gene symbol	*siRNA construct*	**Rel. rate of prion propagation** *FC*	*SD*	*t-test*
Fn1	*siRNA-Fn.1*	2.30	0.30	9.2×10^{-10}
	siRNA-Fn.2	2.36	0.40	6.2×10^{-10}
Iqgap2	*siRNA-Iqgap2.1*	1.74	0.31	5.8×10^{-5}
	siRNA-Iqgap2.2	1.52	0.21	2.6×10^{-3}
Chga	*siRNA-Chga.1*	1.46	0.27	8.2×10^{-3}
	siRNA-Chga.2	1.81	0.26	1.4×10^{-5}
IL11ra1	*siRNA-IL11ra1.1*	1. 65	0.15	2.4×10^{-4}
	siRNA-IL11ra1.2	0.92	0.12	1.2×10^{-1}
Lrrn4	*siRNA-Lrrn4.1*	1.84	0.19	5.2×10^{-6}
	siRNA-Lrrn4.2	1.80	0.26	1.5×10^{-5}
Micalcl	*siRNA-Micalcl*.1	2.22	0.38	7.8×10^{-9}
	siRNA-Micalcl.2	0.95	0.20	6.4×10^{-1}
Igsf5	*siRNA-Igsf5.1*	1.54	0.18	1.8×10^{-3}
	siRNA-Igsf5.2	1.91	0.28	2.1×10^{-6}
Papss2	*siRNA-Papss2*.1	2.26	0.23	1.0×10^{-8}
	siRNA-Papss2.2	1.18	0.16	3.3×10^{-1}
Galt	*siRNA-Galt.1*	1.47	0.29	7.4×10^{-3}
	siRNA-Galt.2	1.32	0.20	6.4×10^{-2}
Rgs4	*siRNA-Rgs4.1*	0.95	0.12	4.5×10^{-1}
	siRNA-Rgs4.2	0.87	0.15	1.1×10^{-1}
Tshz1	*siRNA-Tshz1.1*	0.70	0.13	4.2×10^{-2}
	siRNA-Tshz1.2	1.45	0.35	1.2×10^{-2}
Nckap1l	*siRNA-Nckap1l.1*	1.23	0.26	2.0×10^{-1}
	siRNA-Nckap1l.2	0.50	0.14	1.5×10^{-3}

Supplementary Table S7: Effects of transient gene silencing in susceptible S7 cells. Layers of 1.5×10^4 S7 cells per well of a 96-well plate were transfected with siRNA against gene candidates expressed in susceptible cells (*Tshz1*, *Nckap1l*) and revertant cells (all other genes) followed by prion infection as described in Methods. Fold changes of the relative rate of prion propagation (FC) ± SD were determined.
