# Supplementary material for: Identification of a gene regulatory network associated with prion replication
Source: EMBO J. 2014 May 19;33(14):1527–47. doi: 10.15252/embj.201387150 (PMC4198050; doi:10.15252/embj.201387150)
Supplement: Supplementary file 16 [file embj0033-1527-sd16.pdf]

Gene symbol	*siRNA construct*	Rel. rate of prion propagation		
		FC	*SD*	*t-test*
Fn1	*siRNA-Fn.2*	2.4	0.42	1.4×10^{-12}
Chga	*siRNA-Chga.2*	3.55	0.41	3.7×10^{-18}
Iqgap2	*siRNA-Iqgap2.1*	2.33	0.86	4.3×10^{-11}
IL11ra1	*siRNA-IL11ra1.1*	2.20	0.43	1.6×10^{-10}
Lrrn4	*siRNA-Lrrn4.2*	2.01	0.45	1.7×10^{-8}
Micalcl	*siRNA-Micalcl.1*	2.64	1.11	1.8×10^{-12}
Igsf5	*siRNA-Igsf5.2*	1.37	0.18	4.5×10^{-4}
Itga8	*siRNA-Itga8.2*	1.56	0.30	1.7×10^{-5}
Papss2	*siRNA-Papss2.1*	2.19	0.35	7.4×10^{-10}

Supplementary Table S8: Effects of gene candidate knockdown on the number of PrP^{Sc}-infected cells in RML-chronically infected cells (iS7). Cells were transiently transfected with siRNA against the specified gene candidates. After three days, cells were counted using a Coulter counter and 2×10^4 cells/well transferred onto Elispot plates and the number of PrP^{Sc}-positive cells was determined. Relative numbers of PrP^{Sc}-positive cells expressed as fold change (FC) to controls (NSC) ± SD for three independent experiments are shown. Statistical significance was computed using a two-tailed Student's t-test.
