# Supplementary material for: Identification of a gene regulatory network associated with prion replication
Source: EMBO J. 2014 May 19;33(14):1527–47. doi: 10.15252/embj.201387150 (PMC4198050; doi:10.15252/embj.201387150)
Supplement: Supplementary file 17 [file embj0033-1527-sd17.pdf]

Gene symbol	*siRNA construct*	**Rel. rate of prion propagation** *FC*	*SD*	*t-test*
Fn1	*siRNA-Fn.2*	2.34	0.27	6.2×10^{-12}
Chga	*siRNA-Chga.2*	2.32	0.40	3.5×10^{-11}
Iqgap2	*siRNA-Iqgap2.1*	1.40	0.41	2.9×10^{-1}
IL11ra1	*siRNA-IL11ra1.1*	1.76	0.49	6.2×10^{-4}
Lrrn4	*siRNA-Lrrn4.1*	1.66	0.43	4.6×10^{-3}
Micalcl	*siRNA-Micalcl.1*	1.21	0.45	7.6×10^{-1}
Chga	*siRNA-Chga.2*	1.29	0.66	2.5×10^{-1}
Igsf5	*siRNA-Igsf5.2*	1.14	0.51	4.5×10^{-1}
Itga8	*siRNA-Itga8.2*	2.17	0.75	1.1×10^{-7}
Papss2	*siRNA-Papss2.1*	2.05	0.60	7.8×10^{-5}
Galt	*siRNA-Galt.1*	1.04	0.36	1.2×10^{-1}

Supplementary Table S9: Susceptibility of R7 cells to 22L prions after transient gene silencing of candidate genes. 1.5×10^4 R7 cells per well of a 96-well plate were transfected with siRNA followed by 22L prion infection (10^{-5} dilution) as described in Methods. Relative rates of prion propagation expressed as fold change (FC) to controls (NSC) ± SD are shown. Statistical significance was computed using a two-tailed Student's t-test.
