# Supplementary material for: Identification of a gene regulatory network associated with prion replication
Source: EMBO J. 2014 May 19;33(14):1527–47. doi: 10.15252/embj.201387150 (PMC4198050; doi:10.15252/embj.201387150)
Supplement: Supplementary file 18 [file embj0033-1527-sd18.pdf]

Gene symbol	siRNA construct	Rel. rate of prion propagation FC	SD	t-test
Fn1	*siRNA-Fn.1*	1.70	0.86	9.3×10^{-3}
Chga	*siRNA-Chga.2*	1.29	0.66	2.5×10^{-1}
Galt	*siRNA-Galt.2*	2.82	2.64	1.1×10^{-4}
	siRNA-Galt.3	1.80	0.88	1.7×10^{-3}
IL11ra1	*siRNA-IL11ra1.1*	2.73	1.51	1.1×10^{-6}
Igsf5	*siRNA-Igsf5.2*	1.41	0.67	1.2×10^{-1}
Itga8	*siRNA-Igga8.1*	1.05	0.53	8.5×10^{-1}
	siRNA-Igga8.2	2.18	1.04	7.7×10^{-5}
Iqgap2	*siRNA-Iqgap2.1*	1.41	0.93	1.4×10^{-1}

Supplementary Table S10: Susceptibility of CAD5 cells to RML prions after transient gene silencing of candidate genes. 1.5×10^4 CAD5 cells per well of a 96-well plate were transfected with siRNA followed by RML prion infection (10^{-5} dilution) as described in Methods. Fold changes (FC) in relative prion propagation rates ± SD were determined.
