# Supplementary material for: Identification of a gene regulatory network associated with prion replication
Source: EMBO J. 2014 May 19;33(14):1527–47. doi: 10.15252/embj.201387150 (PMC4198050; doi:10.15252/embj.201387150)
Supplement: Supplementary file 19 [file embj0033-1527-sd19.pdf]

Cell line	Epitope	PCC	M1 (HS epitope)	M2 (ICSM 18)
**S7**	JM403	-0.01 ± 0.02	0.21 ± 0.03	0.27 ± 0.08
**iS7**	JM403	0.03 ± 0.03	0.21 ± 0.06	0.23 ± 0.05
**S7**	10E4	0.07 ± 0.18	0.09 ± 0.05	0.22 ± 0.10
**iS7**	10E4	0.07 ± 0.05	0.16 ± 0.03	0.29 ± 0.06

Supplementary Table S11: No colocalisation of PrP^{C} with HS epitopes 10E4 and JM403. S7 and iS7 cells were co-labelled with conjugated ICSM18 and HS antibodies 10E4 and JM403 as described in Figure 8. Levels of colocalisation, expressed as PCC and colocalisation coefficients M1 and M2, were analysed by Volocity software as described in Methods. Mean values ± SD from eight fields are shown.
