# Supplementary material for: Identification of a gene regulatory network associated with prion replication
Source: EMBO J. 2014 May 19;33(14):1527–47. doi: 10.15252/embj.201387150 (PMC4198050; doi:10.15252/embj.201387150)
Supplement: Supplementary file 20 [file embj0033-1527-sd20.pdf]

Abbreviation shRNA conctruct	***Sequence shRNA of sense strand***
shRNA-Galt.1	AGGATCCGCAACTGTCTATGAA
shRNA-Galt.2	CCGGGCAGATGTGACATCAATA
shRNA-Galt.3	TGGTACAAAGGACCTGTGAAAT
shRNA-Galt.4	TTGGGTAAAGGCTAAGTGTGAC
shRNA-Galt.5	GAGCCATGATGGGCTGTTCTAA
shRNA-Galt.6	TGGTACCTTTCTGTTTGACAAT
shRNA-Iqgap2.1	CGGATGTAATCTCACACAGAAA
shRNA-Iqgap2.2	ACGCTGGAGGAAGAAATCAAAT
shRNA-Iqgap2.3	GAGGAAAGAACTGGAGAAGTAC
shRNA-Iqgap2.4	ATCAATTAAACTGGATGGAAAA
shRNA-Iqgap2.5	TTCCCTCGATCTGCTGCCTTAT
shRNA-Iqgap2.6	GTCAGATCAACTCCAACCAAAG
shRNA-Slc26a4.1	CGGAAGAGCCAGAGGAACTTAA
shRNA-Slc26a4.2	AACCAAAGAAATTGAGATTCAA
shRNA-Slc26a4.3	AGGGGTGAAGATCCTGAGATTT
shRNA-Slc26a4.4	GTGACATCATCTCCGGAGTTAG
shRNA-Slc26a4.5	GTGATATTGGCTCATCAAATAA
shRNA-Chga.1	GGACACTATGGAGAAGAGAAAG
shRNA-Chga.2	GTCCAGAGAATGGGAGGACAAG
shRNA-Chga.3	GGGCCGGGAGCTGGAACATAAG
shRNA-Chga.4	AGGAGCGTCTGTCCAGAGAATG
shRNA-Chga.5	CCGACTCGCTGTCCAAACCCAG
shRNA-Id4.1	AGCTGTGCCTGCAGTGCGATAT
shRNA-Id4.2	CGGCCTGGCTCTTAATTTGCTT
shRNA-Id4.3	ACCAGTATAGACTCGGAAGTAA
shRNA-Id4.4	CAGAGCAGAAATTAAGAGAAAC
shRNA-Id4.5	GAGCAGAAATTAAGAGAAACAA
shRNA-Id4.7	GGCGCTGTGCCTGCAGTGCGAT
shRNA-Id4.8	GGCCGGCGCCGTGAACAAGCAG
shRNA-IL11ra1.1	CGCTCAAGTTCCGGTTGCAATA
shRNA-IL11ra1.2	TTGGAGCAAGTAGCTGTGTTAG
shRNA-IL11ra1.3	GAAGTGTGAAGAGTTGAAATAA
shRNA-IL11ra1.4	CTTGGAGGAAGTGATAACAGAT
shRNA-IL11ra1.5	ACCTGACTCTGGGTTAGGACAC
shRNA-IL11ra1.6	CCTACTGGATGTGAGATTACAG
shRNA-Lrrn4.1	GAGGAACCAGGAAGAGGGATAT
shRNA-Lrrn4.2	AGGGATATCATTTGAGAAGTAA
shRNA-Lrrn4.3	GTCCGTCAGTCTCTTTGGCAAC
shRNA-Lrrn4.4	CAGTCACAACCTGCTGACCGAG
shRNA-Fn1.1	CCCTATCACAGGGTATAGAATT
shRNA-Fn1.2	CGGGAAGCACTATCAGATAAAT
shRNA-Fn1.3	GAGAACAAAGACAGAGACAATC
shRNA-Fn1.5	ACTTCTGAATCTGTAACTGAAA

shRNA-Fn1.6	GCCAGTTTCCATCAATTATAAA
shRNA-Micalcl.1	AGGAGAAGATACTCAGAAGAAG
shRNA-Micalcl.2	AACTCCGAAGCTGGAAAGAAAA
shRNA-Micalcl.3	TTCCCTTGGCCTCAAAGATAAA
shRNA-Micalcl.4	GCCACTCACAGTCCAAAGACAG
shRNA-Rgs4.1	CCCTTCTAAACTAAGTCCCAAA
shRNA-Rgs4.2	CGCCAAGAAGAAGTCAAGAAAT
shRNA-Rgs4.3	CGCAGTCATTGCTCCAGTTTAA
shRNA-Rgs4.4	TAGTAACTTGCCAGAGGGTAAG
shRNA-Rgs4.5	GTCGGAATACAGCGAGGAGAAC
shRNA-Rgs4.6	ATCAAGTCACCTTCTAAACTAA
shRNA-Rgs4.7	CACCATGAATGTGGACTGGCAG
shRNA-Papss2.1	TGAGACAAAGAAAGACCTATAT
shRNA-Papss2.2	GTGGAAAGTGTTGACAGATTAC
shRNA-Papss2.3	ATCTTTCCATCTCCTATGTTAT
shRNA-Papss2.4	GCCTCTGGAGCTCAAACAGAAG
shRNA-Igsf5.1	CAAGACAGTATTGGAGAGGAAG
shRNA-Igsf5.2	ATCATAGAAGGTCCTCAGAATG
shRNA-Igsf5.3	CTCACTTCAACTGCACCGTGAC
shRNA-Igsf5.4	TCCTCAGAAGGTCAGAAATGTG
shRNA-Bambi.1	ACCCGTAAATCTGGTTTCAATA
shRNA-Bambi.2	CGCACAGATTCTACAGTCAAAT
shRNA-Fst.1	ACCGCTGCCAGGTCCTGTATAA
shRNA-Fst.3	CTCGGAAGAAACGGAGGAAGAG
shRNA-Fst.4	TGGGAGAGAGGATGAGAGAGAC
shRNA-Fst.5	ATGAGGGAAAGTGTATCACAAA
shRNA-Fst.6	TGCTGCTACTCTGCCAGTTCAT
shRNA-Dlc1.1	ACCGGAGCCTCAGCACCTGTAA
shRNA-Dlc1.2	CCGTGACGGAGCAGAACTATAA
shRNA-Dlc1.3	CGGACTCCAAGGTGATTGAAAT
shRNA-Itga8.1	CGCTCAGTGCCTTCTTAAGAAA
shRNA-Itga8.2	CGCATTAACTTCGATCTCCAAA

Supplementary Table S12: List of sequences of 22nt sense shRNAs (5’-3’ orientation) used in this study.
