# Supplementary material for: Identification of a gene regulatory network associated with prion replication
Source: EMBO J. 2014 May 19;33(14):1527–47. doi: 10.15252/embj.201387150 (PMC4198050; doi:10.15252/embj.201387150)
Supplement: Supplementary file 21 [file embj0033-1527-sd21.pdf]

Supplementary video Aberrant PrP^d colocalises with NCAM at the ECM. Fixed chronically infected iS7 cells, treated with acetone and GTC as described in Methods were co-labelled with anti-PrP and anti-NCAM, followed by highly cross-absorbed secondary antibodies Alexa Fluor 488 anti-mouse and Alexa Fluor 568 anti-rat antibodies, respectively. Sequences of confocal images, collected at 0.64 μm intervals are shown.
