# Supplementary material for: Identification of a gene regulatory network associated with prion replication
Source: EMBO J. 2014 May 19;33(14):1527–47. doi: 10.15252/embj.201387150 (PMC4198050; doi:10.15252/embj.201387150)
Supplement: Supplementary file 23 [file embj0033-1527-sd23.pdf]

THE
EMBO
JOURNAL

Identification of a gene regulatory network associated with prion replication

Masue M. Marbiah, Anna Harvey, Billy T. West, Anais Louzolo, Priya Banerjee, Jack Alden, Anita Grigoriadis, Holger Hummerich, Ho-Man Kan, Ying Cai, George S. Bloom, Parmjit Jat, John Collinge, Peter-Christian Klöhn

Corresponding author: Peter-Christian Klöhn, UCL Institute of Neurology

Review timeline:

Submission date:	14 October 2013
Editorial Decision:	14 November 2013
Revision received:	13 March 2014
Accepted:	15 April 2014

Editor: Karin Dumstrei

Transaction Report:

(Note: With the exception of the correction of typographical or spelling errors that could be a source of ambiguity, letters and reports are not edited. The original formatting of letters and referee reports may not be reflected in this compilation.)

1st Editorial Decision 14 November 2013

Thank you for submitting your manuscript to The EMBO Journal. Three referees have now reviewed your manuscript and their comments are provided below.

As you can see, the referees find the analysis interesting and insightful, but also find that further analysis is needed to consider publication here. In particular, additional experiments to support that the observed findings are also relevant in the context of infected animals and some better understanding of how ECM proteins modulate prion susceptibility is needed. I know that these two questions are not straightforward to address and I don't expect you to resolve the mechanism in full, but some additional analysis along these lines would be clearly strengthen the paper. Should you be able to address the concerns raised then I would like to invite you to submit a revised manuscript. We normally allow 3 months for revision time, but I can extend that to 6 months if needed. Also if you have any question regarding any of the experiments please don't hesitate to contact me.

When preparing your letter of response to the referees' comments, please bear in mind that this will form part of the Review Process File, and will therefore be available online to the community. For more details on our Transparent Editorial Process, please visit our website: http://www.nature.com/emboj/about/process.html

Thank you for the opportunity to consider your work for publication. I look forward to your revision.

\--

REFEREE REPORTS

Referee #1:

Marbiah, Harvey et al. present work comparing the gene expression differences between cells susceptible and resistant to prion infection. They identify a number of ECM proteins that appear to impact on the rate of prion replication. The manuscript represents an impressively extensive body of work that is likely to be of interest and significance with respect to prion propagation. However, the manuscript is dense and at times difficult to follow; perhaps this is an inevitable consequence of the inherent complexity of the analyses. In my opinion, it would be helpful to show more of the data in the main manuscript and less in the supplement. In addition, the discussion sometimes seems like a series of informational tidbits about different proteins which might benefit from a more cohesive discussion of the impact and importance of the associated experimental data.

1) The details of the SCA assay and determination of TCIUs are unclear. Despite being already published, a concise and clear summary should be included in the materials and methods.
2) Throughout the manuscript there is inconsistent and sometimes confusing use of nomenclature. E.g. PrPd vs PrPSc. The assays used are based primarily on the detection of PrPSc using PK resistance, yet the authors stress the importance of PK-sensitive PrP in infectivity and thus refer to a PrPd species. While the heterogeneity of PrP detected with confocal imaging could be a discussion point, use of more consistent and clearer nomenclature would be helpful.
3) Pg. 7 - "the revertant cells showed a less differentiated morphology than prion-susceptible cells". This is a subjective statement suggesting dedifferentiation of the revertant subclones. It would be helpful to have more discussion of their criteria for such an evaluation and bright field images illustrating their conclusions.
4) Figure S2f is missing
5) It would be helpful to see select supporting data for Table 2, similar to that shown for S2 for PrP.
6) P. 7 and Discussion: The authors should note that previous studies have described the impact of differentiation factors (i.e. NGF) on the prion susceptibility of cells (see R. Rubenstein et al., J Gen Virol, 1990), which are consistent with the effects that they have seen with RA treatments.
7) Often what the authors describe as staining of the ECM in various figures looks like intracellular staining. A clearer description in the Results of how the "ECM level" was identified and differentiated from intracellular staining would be helpful.
8) Fig 3a and associated text: It is not clear to me how the Bodipy staining relates to cholesterol here.
9) P. 22: The word "Revelation" in the heading should be changed to something less religious, like "Detection".

Minor comments:
1) On pg. 5, a sentence reads i.e. expression differences unrelated to the relevant phenotype prion susceptibility. Should this read "of prion susceptibility"?
2) In Figure 1a resistant is misspelled.

Referee #2 :

ECM paper.

This paper describes a technically elegant set of experiments that is largely based upon the prior discovery of highly sensitive prion susceptible cells. The paper points to an ability of the extracellular matrix to impact prion replication in cultured cells, and is comprehensive in terms of the different technologies applied to the paradigm, and the extent to which these individual technologies are carefully validated. The work examines just one type of prion isolate ("strain"), a drawback. Also the central findings of the work apply just one of many types of prion-infected cells (N2a derivatives). The paper contains a potential technical innovation to do with immuno-histochemistry of ECM-associated abnormal PrP but this is limited to tissue culture cells. Overall the authors might have an important insight into a signature of infected cells but the paper falls short

of demonstrating how this might be of relevance in the context of infected animals - an experiment to address this gap would greatly improve the paper.

Main Comments.

The paper is at times challenging to read, an effect that is compounded by the large amount of data presented; at times in the RNA data it is hard to keep track of what is going down, what is going up and if the data are diverging from or falling outside the core hypothesis being presented.

"sus versus rev" is jargon and it would be useful to understand which is the numerator without trawling through all the Tables. I think the authors mean over expressed in revertants versus susceptible cells.

The effect with retinoic on replication is interesting, as is the concept that the revertants are less differentiated, and then the question arises as to whether this concept about differentiation and the ECM is followed through by the behavior of all the transcripts. If reversion is associated with less differentiation but retinoic acid with more differentiation then the genes down-regulated in revertants might be expected to be reinstated by RA. The reciprocal idea that genes up-regulated in revertants are down-regulated by RA. These relationships are presented in Table 1b with a set of 18 convergent genes, as well as 90 or so other genes. The latter type of behavior is demonstrated by the performance of Id4 (+3.4, then -5.1) and follistatin (+2.8 then -3.2) but then again 30 or so other genes in Fig S5a seem to be deviating from this behavior. The same sort of comment can be applied to Fig S5b where two genes are highlighted out of 53. While it could be said that these other genes are not of interest because they fall outside of the GO Term classification scheme, it is noticeable that some more strongly regulated genes are excluded (IGfbp5, Cyp26b1, Csnk, Ifi203, Mmp15, Matn 2). Also, there may also be issues of curation errors e.g. it is odd that a gene such as IgfBp5 does not fall under the category of development or cell regulation, and that Mmp5 and Matrilin 2 are not of interest when the title of the paper includes the term "extracellular matrix". Is there a reason for this?

Minor points:

Since EMBO J asks about title "Prion replication in cultured cells is controlled by..." would be nearer the mark because the current title suggests a fundamental relationship that would be true in the simplest model system and generalized, i.e. non-genetically engineered animals infected with more than one type of prion strain. This is not yet established.

While at first glance the paper seems to be describing a totally new area, the literature coverage is not adequate. There are certainly prior reports of the intersection of prion biology with that of the ECM (and fibronectin) that are notably absent from the Discussion (e.g. Schachner, Prusiner, Schmitt-Ulms work on NCAMs and adhesion molecules; studies with RGD peptides).

R33 and NN2a are not represented on the heat map.

In tables S1 and S2 it would be useful to color-code the genes that are going to appear in other compilations.

Table S3 order of presentation does not match the text.

"epigenetic relationship" p5. Has connotations of methylation differences, genealogy or kinship may be better to use.

RA abbreviation is used first in Table 1 and should be explained at that time

The gene WWTR1 is described as being expressed in revertants, but is not listed in Table S2 and hence there is no value for the fold change of this transcript.

Referee #3 :

This manuscript addresses the question of why there are significant differences between cell lines in their susceptibility to infection by prions. Using a transcriptomic approach, the authors identified a list of genes that are differentially expressed in prion-resistant and -susceptible clones of N2a neuroblastoma cells, and in resistant clones that have been rendered susceptible by treatment with retinoic acid. Several of the identified genes have a role in extracellular matrix remodeling, and knocking these down by RNAi or shRNA turned resistant cells into susceptible cells. Overall, the authors conclude that extracellular matrix proteins play an important role in prion replication.

This study addresses an interesting and important question in prion biology, namely what controls the susceptibility of cells to prion infection. There is a wide variability in prion susceptibility among different cell lines, and even among different clones of the same cell line, but the factors that control this phenomenon are poorly understood. The manuscript is well written and the data are presented and discussed in a clear and logical way. Using a dual-pronged transcriptomic analysis to find the overlap of genes involved in susceptibility is a clever approach, and the experiments to confirm the functional role of the identified gene candidates by RNAi/shRNA-mediated knock-down nicely confirms the hits.

However, there are several important issues that need to be addressed by the authors:

1. Perhaps most importantly, the authors do not explore in a mechanistic way how these gene products, which lie in the extracellular matrix, could be modifying prion susceptibility or propagation. Is it because they block access of PrPSc to surface PrPC? Do they inhibit the conversion process itself, as some extracellular glycosaminoglycans are thought to do? Why does such a diverse collection of proteins all seem to have the same effect? The RGD experiment (Fig. 5) makes an initial foray into the underlying mechanisms, but to address these questions adequately would require further experimentation.

2. The cell biological evidence for the localization of some of the candidate proteins in the extracellular matrix is not convincing. Fibronectin and integrins are well-characterized matrix proteins, but Chga, lqgap2, Micalcl, papss2, and Galt are not. Indeed, it is not clear why these proteins would accumulate extracellularly. In the images shown in Fig. 2, for example, how can the authors discriminate between proteins in the extracellular matrix, and those that are closely associated with the extracellular or intracellular surfaces of the plasma membrane? The authors should try treatments that disrupt the extracellular matrix, or isolate subcellular fractions enriched in ECM to address these points.

3. The authors should provide additional evidence for their hypothesis that ECM proteins regulate prion replication by trying other treatments to disrupt the ECM, for example incubation with heparitinase or chlorate (to inhibit GAG sulfation) to see if these inhibit PrPSc generation.

4. The authors have shown that knock-down of the expression of the candidate genes in resistant cells renders them prion-permissive. To confirm their results, they should also demonstrate that over-expression of these genes turns susceptible cells into resistant cells.

5. The authors should do a better job distinguishing whether the candidate genes control prion susceptibility vs. prion propagation. In other words, do the encoded proteins block initial infection, or slow ongoing infection? A revision of the title should be considered if the two possibilities can't be distinguished. In this regard, a number of previous studies have shown that GAGs modulate prion replication. The authors should discuss more extensively how these earlier results relate to the results in this paper.

Minor points:

6. It is not easy to find panel F in Figure S2. The authors should rearrange the panels in this figure.

7. In Figure 2, the authors should plot the number of cells positive for each protein before and after RA treatment (analogous to panel E, which plots this for sensitive vs. resistant cells).

8. In Figure 3A, the authors should explain what Bodipy is, and how is it being used in this experiment.

9. For Figure 5D, the authors should explain how knock-down of the MMPs was performed. This is not stated in the legend or the Methods section.

1st Revision - authors' response 13 March 2014

Reply to reviewers
Reviewer #1

We thank the reviewer for the positive comments and have now followed this reviewer's suggestion to present more data in the main body of the manuscript. We are now presenting the maximal number of figures, i.e. nine figures. Coherence and readability of the discussion has also been greatly improved.

Major points:

(1.) As suggested, a descriptive summary of the detection of infected cells using the Scrapie Cell Assay (SCA) has now been added to the methods section, 'Quantification of prion infection and rates of prion replication'.

(2.) This reviewer notes that the nomenclature used of disease-associated PrP is confusing. Whilst indeed the field is in general inconsistent in the use of terms for disease-associated forms of PrP, we have defined, in the introduction, the terminology used in this manuscript (page 4, 3rd paragraph). For clarity, the term PrP^{Sc}, operationally defined as proteinase K-resistant (PK) PrP, is exclusively used to report SCA data, where PrP^{Sc} is the experimental readout, whereas the term 'disease-associated PrP' (PrP^{d}) is used consistently to describe deposits of abnormal PrP in immunohistochemistry, since we cannot say whether it is PK resistant.

(3.) We agree with this reviewer that brightfield images are helpful to depict the morphological difference between revertant and susceptible clone, as described on page 7, 1st paragraph. We have now added a set of brightfield images (Supplementary Figure S1) and explain morphological differences between the cell types in the legend. Briefly, susceptible cells are characterised by long, neurites that generally form branches and networks with neurites from other cells, whereas revertants show a less differentiated phenotype with short, mostly unbranched neurites.

(4.) As suggested we have now rearranged the panels in Figure 3 (was Supplementary Fig S2 in previous version) for clarity.

(5.) The reviewer suggests adding supporting data to Table 2 (now Table 1). We would like to remark that, as noted in results (page 9) the full data set for the validation of gene candidates with all tested shRNAs is documented in Supplementary Table S6, a data set which summarises about twenty different experiments. We therefore do not think that deconvoluting this already comprehensive Supplementary Table adds value to the manuscript.

(6.) We are grateful for the reviewer's suggestion to include a previous report (Rubenstein et al., J Gen Virol, 1990) as supportive evidence for the link between differentiation and prion susceptibility (page 7, 2nd paragraph).

(7.) This reviewer suggested to include a description of how proteins, expressed at ECM level were identified during laser scanning microscopy. We have now added a small paragraph in Methods, section under 'Analysis of confocal images'. Furthermore, where images of proteins, expressed at plasma membrane or in the cytosol, and distinct from the ECM are shown, a clear reference in the legend has been added.

(8.) The reviewer suggested specifying how BODIPY staining relates to cholesterol. For clarity, we now explain in results (section 'Detection of aberrant PrPd deposits at the ECM after delipidation with acetone') that BODIPY 500/510 is a fatty acid analogue, which is deposited in triacylglyceride rich lipid droplets. We further confirmed this result by using BODIPY-cholesterol, a lipid analogue that mimics trafficking and deposition of cholesterol (Supplementary Figure S4).
(9.) We are grateful for the reviewer's suggestion to replace 'revelation' with 'detection'.

Minor points:
The two errors mentioned were corrected as suggested.

Reviewer #2

We are very grateful for the complimenting words regarding our approach to identify prion modifier genes. We welcome this reviewer's suggestion to investigate whether the observed effects are strain-dependent. We have infected revertant R7 cells with 22L, a prion strain that shows distinct PrP^{Sc} deposition in mouse brains, when compared to RML {Karapetyan, 2009 633 /id} (Supplementary Table S8). Whilst a trend to increased prion propagation rates was observed for all genes studied, except for Galt, statistically significant results were obtained for more than half of the genes, including *Fn1*, *Itga8*, *Papss2*, *Chga*, *Il11ra1* and *Lrrn4*. We therefore conclude that some of the identified genes may control prion susceptibility in a strain-independent manner. We have added this data to the results section on page 10, 2nd paragraph.
We are grateful for the suggestion of this reviewer to test whether these finding are limited to N2a-derived cells. To address this question we silenced a selection of 7 genes in CAD5 cells, a cell line derived from CNS catecholaminergic-differentiated (CAD) cells {Mahal, 2007 15299 /id}, followed by infection with mouse RML prions (Supplementary Table S10). Knockdown of four out of eight candidate genes (Fn1, Galt, Il11ra1, and Itga8) resulted in a significant increase in susceptibility. This result demonstrates that these findings are not exclusive for N2a cells. This paragraph was added to the manuscript on page 10, 3rd paragraph.
This reviewer suggested we provide supporting evidence for a role of the identified gene signature in infected animals *in vivo*. We completely agree that this is an important question to investigate and preliminary experiments to investigate this are in progress. However, scrapie incubation times in mice are typically 150-300 days and we expect that a systematic *in vivo* validation of our gene candidates will take at best 3-4 years to accomplish. We therefore feel that this suggestion is far beyond the scope of this manuscript.

Major points

As suggested by this reviewer we have improved consistency of reporting in regards to expression differences in susceptible and revertant cells. Consistent with Figure 2 (previously Table 1) we have now denoted genes expressed in susceptible cells with '-' and gene expressed in revertants with '+'. Where gene expression differences are reported in supplements, we have added titles to all tables to clearly denote whether genes are up- or downregulated. All abbreviations in Figure 2 (previously Table 1) are now specified in the legend.
This reviewer points out that genes that are strongly regulated by retinoic acid (RA) treatment in R7 cells, like IGfbp5, Cyp26b1, Csnk, Ifi203, Mmp15, Matn 2 have not been considered for gene validation, even though they fall into the GO categories 'development' and 'extracellular matrix'. I would like to respond to this important question by reiterating the approach of our gene validation triage. The observation that RA treatment of revertants led to a marked gain of susceptibility (Supplementary Table S4) prompted us to test the hypothesis that cell differentiation is associated with susceptibility to prion propagation. We therefore investigated whether genes differentially regulated by RA are found in the previously acquired list of genes differentially expressed between susceptible and revertant cells (Supplementary Table S5). Remarkably, 18 gene candidates were found in both gene candidate lists (Supplementary Tables S2 and S5). Since both expression studies are independent and chances that a gene falls by chance into both candidate lists highly unlikely, i.e. $< 4x10^{-5}$ (2 lists of 200 genes, and assuming 30,000 genes represented in mouse genome), we thus experimentally validated the identification of 18 genes which are controlled by the differentiation state of cells and associated with susceptibility. For clarity we have now replotted the incremental increase in susceptibility of revertants upon RA treatment (Supplementary Table S4) and added this graph to Figure 2 (Figure 2A).

Minor points

The reviewer suggests amending the title to 'Prion replication in cultured cells is controlled by expression of extracellular matrix proteins' to indicate that this study represent *in vitro* data. I have now changed the title to "Identification of a gene regulatory network associated with prion replication", a concise title, which I think represents best the gist of our study.
This reviewer suggests discussing work by Schachner, Prusiner, and Schmitt-Ulms on the role of NCAMs and adhesion molecules in prion propagation. Whilst I would greatly appreciate to extend the discussion, we are strictly limited by the number of words and references, and given the breadth of data we are providing there is no scope for a broader discussion of our findings. This prompted us to limit the discussion to most relevant publications in the context of our study.
R33 and N2a cells have not been considered in the heat map for reasons discussed in the first chapter of results, 'isolation of cognate prion-resistant revertants from highly susceptible cells'. Briefly, the global gene expression profiles of R33 and N2a cells are vastly different from PK1 subclones (Fig. 1D) and were therefore excluded from analysis.
I am grateful for pointing out a reference error for Table S3. This has now been rectified.
Further, the term 'epigenetic relationship' has now been replaced with 'kinship' and the abbreviation RA has now been defined in the legend of Figure 2.

Reviewer #3
We thank this reviewer for the positive comments.

Major points
(1.) We thank this reviewer for suggesting to further explore the underlying molecular mechanisms by which protein candidates, identified in our study and located at the extracellular matrix (ECM), affect prion replication. In this context, the reviewer suggests investigating whether the candidates "inhibit the conversion process itself, as some extracellular glycosaminoglycans are thought to do".
We now present evidence that silencing of Papss2 (3'- phosphoadenosine-5'-phosphosulfate (PAPS) synthase

2), one of the principal enzymes required for the sulfation of extracellular matrix molecules, leads to ablation of heparan sulfation with a concomitant increase in prion propagation (Figure 8). Similar effects were shown by incubation of cells with sodium chlorate, an inhibitor of sulfurylase, an enzyme required for PAPS formation (Figure 8). These results suggest that the sulfation state of HSPGs is negatively correlated with prion replication. Notably, we show that *Papss2* knockdown leads to higher levels of deposited PrP^C at the ECM (Supplementary Figure S6 and Figure 9). A similar effect was observed for *Fn1* knockdown (Figure 9). Under these conditions PrP^C protein levels were shown to increase significantly on Western Blot (Figure 9). We suggest that these perturbations of PrP^C deposition are due to perturbations in ECM homeostasis and may facilitate the formation of seeds for prion conversion. We further discuss the relevance of this finding in the context of published data in the discussion.

(2.) This reviewer argues that genes like Chga, Iqgap2, Micalcl, Papss2 and Galt have not been shown to localise in the ECM and suggests a complex set of experiments, like subcellular fractionation to further elucidate the location of proteins.
We appreciate the suggestions, but we do not state in the manuscript that all protein candidates are localised at the ECM. Whilst we present evidence for the location at the ECM of Fn1, Chga (Figure 4), Micalcl (Supplementary Figure S3), we show that other protein candidates like Lrrn4, Il11ra1 (Figure 4) and Iqgap2 (Supplementary Figure S3) are expressed at the membrane. As mentioned previously (reviewer #1) we have now included a paragraph in Methods (section 'analysis of confocal images') how proteins expressed at ECM level were identified by laser scanning microscopy.

(3.) This reviewer suggests using treatments to disrupt the ECM and to inhibit GAG sulfation, like heparitinase and chlorate, respectively, to test effects on prion propagation.
As mentioned above, chlorate treatment of cells greatly reduced sulfation of HSPGs and increased conversion, in agreement with effects shown upon *Papss2* knockdown. Whilst these results clearly demonstrate an association between the sulfation state and prion conversion rates, I do not believe

the enzymatic disruption of the ECM by heparinases or chondroitinase will help to address molecular mechanisms of conversion, since such treatments affect a myriad of extracellular proteins and receptors.

(4.) The reviewer suggests overexpressing gene candidates to test whether susceptible cells can be rendered resistant.
To address this question we cloned MGC (Mammalian Gene Collection) clones for all candidate genes into Gateway-adapted vectors to express genes after transfection and retroviral gene delivery, respectively. However, whilst HEK293 cells showed elevated expression levels after gene delivery, we were not able to increase expression of candidate genes in prion-susceptible cells, whilst enrichment of overexpressing cells by FACS was prohibited, since green fluorescent protein is absent in these vectors.

(5.) We are grateful for the suggestion of this reviewer to better differentiate whether the encoded proteins inhibit initial infection or the ongoing infection.
We have addressed this question by silencing identified gene candidates in chronically prion-infected cells, alongside with scrambled RNA controls (Supplementary Table S8). Remarkably, a significant increase of prion conversion rates was observed for all genes. This suggests that the identified gene regulatory network affects prion conversion. These results have been included in results, section ‘Identification of a gene regulatory network associated with prion propagation’.

Minor points
(6.) Panel F in Supplementary Figure S2 (now Figure 3) was difficult to find and has now been rearranged for clarity.

(7.) We are grateful for the suggestion of this reviewer to include the number of cells positive for each protein before and after RA treatment. These analyses have now been added to Figure 4.

(8.) The use of C1-BODIPY 500/510 to monitor the delipidation of cells has now been described in results section ‘Detection of aberrant PrP^d deposits at the ECM after delipidation with acetone’. We furthermore confirmed these results with a distinct BODIPY analogue, BODIPY-cholesterol (Supplementary Figure S4).

(9.) As suggested details about gene silencing of MMP2 and MMP9 have now been included in the legend of Figure 7.

Pre-Decision letter 9 April 2014

Thank you for submitting your revised manuscript. Your study has now been seen by the two referees and their comments are provided below.

As you can see both referees appreciate very much the introduced changes and support publication here. I am therefore very pleased to accept the paper.

There are just a few minor things to fix:

- Referee #2 suggests to add a minor discussion point - please see below.
- Please add the accession number for the microarrays
- Please add Author Contributions (right after acknowledgements and before COI)

You can send me a modified text file by email. Once we get that resolved we will transfer the paper to our publisher.

Congratulation on a great paper!

REFEREE REPORTS:

Referee #1:

The authors have attended to the comments and the corresponding revisions in a most thorough manner. I approve of the new title. I have no further comments on the paper.

Referee #2:

The authors have done a superb job of addressing the concerns of all the reviewers.
I am particularly intrigued by the new experiments looking at PrPC levels at the membrane after silencing of Papss2 and treatment with chlorate. In light of this result, I would suggest one more minor addition to the Discussion: Do the authors think that a simple increase in PrPC levels (as shown by Western blot) is sufficient to explain the increase in prion replication?

minor point:

P.15, line 5, instead of "As shown in figure 8" the authors should say "As shown in figure 8F".

© European Molecular Biology Organization
